# Anion exchange chromatography-based purification of plant-derived nanovesicles from *Brassica oleracea* L.: molecular profiling and bioactivity in human cells

**DOI:** 10.3389/fbioe.2025.1617478

**Published:** 2025-07-31

**Authors:** Clarissa Zanotti, Simona Arena, Sabrina De Pascale, Valentina Ciaravolo, Rosalia Ferracane, Antonio Dario Troise, Chiara Pontecorvi, Francesca Pacello, Chiara Niespolo, Angelo Gismondi, Andrea Scaloni, Mauro Marra

**Affiliations:** ^1^ Department of Biology, University of Rome Tor Vergata, Rome, Italy; ^2^ PhD Program in Cellular and Molecular Biology, Department of Biology, University of Rome “Tor Vergata”, Rome, Italy; ^3^ Proteomics, Metabolomics and Mass Spectrometry Laboratory, ISPAAM, National Research Council, Portici, Italy; ^4^ Arterra Bioscience SpA, Naples, Italy

**Keywords:** plant-derived nanovesicles, *Brassica oleracea* L., proteomics, lipidomics, miRNomics, wound healing, cross-kingdom regulation

## Abstract

Plant-derived nanovesicles emerge as a promising alternative to mammalian-derived exosomes with distinct advantages, including lower immunogenicity, enhanced bioavailability, and the presence of bioactive plant metabolites. They have been shown to cross biological barriers, delivering therapeutic molecules that modulate gene expression, inflammation, oxidative stress, and cancer-related pathways. However, challenges remain that limit applicative use, including poor knowledge of their interactions with mammalian host cells and primarily the lack of a cost-effective and scalable method to obtain highly purified plant-derived nanovesicles. To address these limitations, we have developed an advanced purification platform that integrates ultrafiltration with anion exchange chromatography in a fast protein liquid chromatography system. This approach was validated using it in the isolation of plant-derived nanovesicles from *Brassica oleracea* L. seedlings, resulting in highly purified and concentrated preparations. Comprehensive molecular analyses, including proteomics, lipidomics, metabolomics, and miRNA profiling, characterized the nature of the purified plant-derived nanovesicles. Furthermore, their wound healing and anti-inflammatory properties were demonstrated *in vitro* and correlated to the potential biological activities of cargo miRNAs species by bioinformatics, highlighting the potential in nanomedicine of anion exchange-purified brassica nanovesicles. This study provides a scalable and efficient purification strategy, which might pave the way for broader applications of plant-derived nanovesicles in the clinical, nutraceutical and pharmaceutical fields.

## 1 Introduction

Extracellular vesicles (EVs) are lipid-membrane delimited vesicles (30–5,000 nm diameter) produced by various organisms, including animals and plants, involved in cell-to-cell communication. They carry, protect, and deliver a great variety of bioactive molecules, including proteins, lipids, nucleic acids and metabolites to target cells (Johnstone et al., 1987; Théry et al., 2002). Recent improvement of purification and molecular characterization methods have led to the identification of a great variety of EVs that differ in dimension, biogenesis and cargo content, highlighting a picture of high heterogeneity and complexity (Jeppesen et al., 2023). Mammalian-derived exosomes (MDEs) are the smallest subset (30–200 nm) of EVs. They originate from the invagination of late endosomes, forming multivesicular bodies (MVBs), which then fuse with the plasma membrane to release exosomes into the extracellular environment (Raposo and Stoorvogel, 2013). Once secreted, MDEs and other EVs travel through the extracellular space and bind to recipient cells via specific adhesion molecules. Upon internalization, MDEs deliver their cargo of proteins, lipids, mRNA, microRNA (miRNA) and metabolites, influencing gene expression and cell functions (Ocansey et al., 2020; Zhang and Yu, 2019). For its therapeutic potential in the treatment of diseases, research on MDEs has grown exponentially since their discovery in 1983 (Pan and Johnstone, 1983). Much less is known regarding plant-derived nanovesicles (PDNVs), despite they have been observed sixteen years before MDEs (Halperin and Jensen, 1967). PDNVs present significant heterogeneity, which poses challenges for their characterization and therapeutic application. This heterogeneity arises from differences in plant species, origin (especially for those derived from tissue homogenization) and isolation methods. Moreover, PDNVs exhibit diverse molecular contents, including varying profiles of lipids, RNAs, and bioactive compounds. Additionally, the lack of well-defined and consistent protein markers across different PDNV populations complicates their identification and standardization (Rodriguez de Lope et al., 2024).

The interest in PDNV research has been driven by the idea that the consumption of certain foods is associated with health benefits and a reduced risk of disease (Sarasati et al., 2023). In fact, PDNVs derived from edible plants have been shown to be absorbed into the mammalian gastrointestinal tract (Mu et al., 2014; Wang et al., 2014) and to deliver various bioactive molecules, including miRNAs, proteins and lipids through diverse biological barriers (Wang et al., 2018). Furthermore, specific plant-derived miRNAs have been found to regulate human genes involved in inflammation, oxidative stress, and cancer, suggesting their role in cross-kingdom communication (Mu et al., 2023; Baldrich et al., 2019; Li et al., 2021). PDNVs share similarities with MDEs in terms of size, morphology, and ability to transport bioactive molecules to target cells. However, PDNVs possess distinct attributes that confer unique biological properties of pharmaceutical interest. These properties include a lower risk of immunogenicity and the presence of bioactive plant secondary metabolites (Raimondo et al., 2015). Accordingly, PDNVs have been proposed in nanomedicine applications as ecofriendly, robust, safe, and cost-effective carriers. In comparison, MDEs are often associated with complications such as immunogenicity, cytotoxicity, and a costly production process (Li et al., 2023). Due to their natural source and composition, PDNVs are low immunogenic, a fact that leads to improved biodistribution and greater bioavailability (Zhang et al., 2017). Furthermore, unlike mammals, plants do not carry zoonotic or human pathogens, making PDNVs a safer choice. Despite a number of studies have shown that PDNVs can function as natural cross-kingdom delivery systems for bioactive compounds, suggesting a range of applications in different fields, including clinical, nutraceutical, cosmeceutical, and agricultural, a gap of knowledge regarding different aspects of PDNVs physiology, biochemistry and pharmacology needs to be filled in order to open the possibility of more focused and effective applications, as well as to expand the potential of PDNVs. For example, the mechanism of interaction of PDNVs with mammalian recipient cells and the endocytic pathway involved in their uptake is unknown, as well as their pharmacokinetic and pharmacodynamic properties in human fluids. Currently, the most significant challenge in developing affordable therapies based on PDNVs is the lack of a standardized, scalable, and cost-effective platform capable of producing homogeneous preparations of PDNVs with high yield. Different methods originally developed for mammalian EVs have been adapted to PDNVs. However, all these methods are unsatisfactory in terms of yield, purity, scalability, and cost-effectiveness (Silva et al., 2023; Liangsupree et al., 2021; Subha et al., 2023). The up to date most widely used technique, differential ultracentrifugation, is limited by contamination of protein/nucleic acid aggregates, which affects vesicle purity, and is also costly and challenging to be scaled up. Ultracentrifugation in density gradients can enhance nanovesicle purity but it also reduces yield. Alternatively, polymer-induced precipitation has been used in many cases. This method is cost-effective and scalable, but the purity of PDNVs is low, because of the coprecipitation of cellular contaminants. A more recent method for obtaining large amounts of PDNVs with good purity is sequential ultrafiltration/tangential flow filtration. It allows to obtain PDNVs of relatively high purity; however, membrane occlusion and potential disruption of PDNVs, due to the pressure applied during filtration, are very common drawbacks of this approach. Another size-based separation method is size exclusion chromatography. This technique is simple, preserves vesicles integrity, but is limited by the coelution of protein aggregates, and results in sample dilution. Novel purification methods based on affinity capture with magnetic beads-linked antibodies have been introduced to precipitate PDNVs from crude extracts. They offer high specificity, allowing the extraction of highly purified nanovesicles, but are limited by their high costs, poor characterization of surface PDNV markers and difficult scalability. Other methods, which still require further characterisation for common use, include affinity capture with biopolymers, microfluidic technologies, and asymmetric flow field flow fractionation (Li et al., 2023; Mu et al., 2023; Shkryl et al., 2022).

Recently, some authors have explored the possibility of purifying MDEs by anion exchange chromatography, taking advantage of the net negative charge normally present on the surface of these vesicles (Silva et al., 2023; Gagnon et al., 2020). Anion exchange chromatography allows the processing of large sample volumes and is both cost-effective and scalable. Indeed, this procedure has been shown to be highly efficient in the purification of human EVs (Silva et al., 2023; Gagnon et al., 2020), thus representing a promising starting point for its adaptation to the purification of PDNVs. With the aim to overcome the above-reported challenges in the preparation of PDNVs, we have developed a purification platform that integrates ultrafiltration (UF) with anion exchange chromatography in a fast protein liquid chromatography (FPLC) system, which provides superior resolution, reproducibility, and automation, making it an ideal choice for scaling up PDNVs isolation. The procedure has been validated by applying it to the purification of PDNVs from a plant source universally appreciated for its nutritional properties such as *Brassica oleracea* L. seedlings, which can be easily and cost effectively grown in large amounts under controlled conditions. The results of purification have demonstrated that the anion exchange-based procedure was efficient, providing concentrated samples of homogeneous PDNVs as far as dimensions and morphology. Purified samples have been extensively characterised as far as their molecular cargo, using proteomic, lipidomic, metabolomic, and miRNomic procedures. Finally, the wound healing capacity and anti-inflammatory activity of purified brassica-derived nanovesicles has also been evaluated *in vitro* on human cell cultures and correlated to the potential biological activities of cargo miRNAs by bioinformatics.

## 2 Materials and Methods

### 2.1 Chemicals

All chemicals (acetonitrile, water, methanol, chloroform, isopropanol, and formic acid) and analytical standards (phosphocholine, and various polyphenols, triglycerides, diglycerides, ceramides and phytochemicals) used in liquid chromatography high-resolution tandem mass spectrometry (LC-HR-MS/MS) analysis were of mass spectrometry-grade and were obtained from Merck (Darmstadt, Germany). Reagents for SDS-PAGE were purchased from Bio-Rad (Hercules, CA, USA). All other chemicals were of analytical grade and were purchased from Merck (Darmstadt, Germany).

### 2.2 Plant materials and growth conditions

Seeds of *B. oleracea* L. *var. Italica* were obtained from Natures Root (www.naturesroot.co.uk). The seeds were pre-soaked in distilled water for 2 h and then evenly distributed on trays (50 × 70 cm) containing a coco-perlite mix moistened with distilled water as growth substrate (Gold label coco perlite 70/30). Twenty gr of seeds were used for each tray. The trays were then placed in a climate chamber (VB1514 Vötsch, Rosenfeld, Germany) at 22°C and 80% v/v humidity, with a 16/8 h light/dark cycle. The aerial part of the seedlings was cut and collected for PDVN preparation after two weeks of cultivation.

### 2.3 Preparation of the PDNV extract for anion exchange-FPLC

One hundred gr of the aerial part of *B. oleracea* L. seedlings were harvested and homogenized with a waring blender in 100 mL of phosphate-buffered saline (PBS) pH 7.4, supplemented with 1 mL of Protease Inhibitor Cocktail (Thermo Scientific) containing 1 mM sodium azide, 100 mM phenylmethylsulphonyl fluoride, and 1 mM leupeptin, to minimize proteolytic degradation during extraction, at 4°C. The homogenate was filtered through a cheesecloth and centrifuged at 8,000 x g and 18,000 x g, for 30 min, at 4°C, to remove cell debris and other contaminants. The supernatant was then sequentially filtered through 40 μm, 0.80 μm, and 0.22 μm membrane filters. After sequential filtration, the sample was concentrated to 5 mL, by tangential flow filtration (TFF/UF), using a 100-kDa molecular weight cut-off ultrafiltration membrane (Amicon Ultra 15, Merck). The sample was then diafiltered against a buffer composed of 50 mM Tris-HCl, 50 mM L-arginine, 5 mM CaCl_2,_ pH 7.5, to perform treatment with micrococcal nuclease (MNase, Thermo Scientific) at a final concentration of 150 U/mL, for 2 h, under gentle stirring, at 37°C. After nuclease treatment, the sample was diafiltered against 50 mM HEPES, 180.7 mM NaCl, pH 7.4 (Silva et al., 2023). The resulting sample was used for anion exchange-FPLC purification. All steps were performed with samples and buffers at 4°C.

### 2.4 Anion exchange-FPLC purification of PDNVs

Anion exchange chromatography was performed using an AKTA FPLC apparatus (Cytiva), equipped with a UV detector set at 280 nm and a 2 mm path length UV cell. Separation was performed on a CIMmultus™ EV monolith column (1 mL volume, Sartorius) according to the manufacturer’s protocol, with minor adjustments. Briefly, the column was equilibrated with 5 column volumes (CVs) of 50 mM HEPES, 20 mM NaCl, pH 7.5. Subsequently, 2 CVs of the pre-purified PDNV sample were injected at a flow rate of 2 mL/min. The column was then washed until stable UV absorbance and elution was performed using a linear gradient from 0% to 100% of 1.0 M NaCl in 50 mM HEPES buffer, pH 7.5, at a flow rate of 2 mL/min, over 20 CVs. Two mL fractions were collected and analyzed by Bradford assay (Bradford, 1976) for protein content determination, and by NTA analysis for PDNVs detection. The PDNV-containing fractions were pooled and stored at 4°C for analysis within 3 days, or frozen at −20°C, until use.

### 2.5 Nanoparticle tracking analysis (NTA)

The size distribution and particle number/concentration of purified PDNVs were analysed by NTA, using Nanosight NS300 (Malvern Instrument, UK). Before measurement, the PDNVs were diluted 100 to 500 times in filtered PBS (0.2 µm), to achieve a final particle concentration ranging between 10^8^ and 10^9^ particles/mL. Each measurement was performed by scanning 11 cell positions each and capturing in scatter mode 60 frames per position. For each sample, three-time intervals of 30 s were recorded and analyzed with Nanoparticle Tracking Analysis analytical software v. 3.4 (Malvern Instrument, UK). The area under the histogram for each sample was analyzed in triplicate; the measurements were averaged and used as one particle concentration measurement. Three independent experiments were carried out. All NTA measurements were performed with identical system settings for consistency. The analysis was performed at the Core Facilities of Istituto Superiore di Sanità, Rome, Italy.

### 2.6 Transmission electron microscopy (TEM)

Negative staining of PDNV samples was performed as previously described (Federici et al., 2020). Briefly, PDNVs (>10^10^ before and >10^7^ sorting, measured by NTA) were suspended in 100 µL of PBS and 10 µL were adsorbed on formvar-carbon coated grids. Five µL of 4% w/v ammonium molybdate, pH 6.4, were added for 30 s, as contrasting solution, and adsorbed with filter paper. The samples were air dried and observed by PHILIPS EM208S TEM (FEI, ThermoFisher) at the Department of Physics, Istituto Superiore di Sanità, Rome, Italy.

### 2.7 SDS-PAGE and western blotting

SDS-PAGE was performed with a Mini Protean II Dual SLAB Cell apparatus (Bio-Rad, Hercules, California, USA) according to Laëmmli (1970). Proteins were visualized by staining with 0.1% w/v Coomassie Blue (Bio-Rad, Hercules, California, USA) dissolved in 50% v/v methanol and 10% v/v acetic acid. SDS-PAGE-resolved proteins were transferred in a Trans Blot semi-dry Transfer cell (Bio-Rad) onto a polyvinylidene fluoride membrane (Bio-Rad), as described previously (Zanotti et al., 2024). The membrane was incubated with anti-TET8 primary antibody (PhytoAB, USA, code n. PHY3337A) at a 1:2,000 dilution, overnight, at 4°C. After incubation, the membrane was washed three times with TTBS and incubated for 1 h with horseradish peroxidase-conjugated secondary antibodies (anti-mouse 1:10,000, Bio-Rad, Hercules, California, USA). The antigen-antibody interaction was revealed with a 1:1 luminol and Peroxide Buffer solution (Euroclone, Milan, Italy). The chemiluminescence signal was acquired with VersaDoc™ 4000 MP (Bio-Rad).

### 2.8 Protein extraction and nanoLC-ESI-MS/MS analysis

PDNVs from three independent *B. oleracea* leaf preparations, each one corresponding to 0.7 mg of proteins, were independently purified by anion exchange-FPLC. Pooled fractions were dried in SpeedVac roto-evaporator (ThermoFisher Scientific, USA) and the corresponding pellets were extracted using 50 μL of lysis buffer containing 7 M urea, 2 M thiourea, 4% w/v 3-[(3-cholamidopropyl)dimethylammonio]-1-propanesulfonate e 1% w/v dithiothreitol, and added with a protease inhibitor cocktail for plant tissues (Merck-Sigma-Aldrich, Darmstadt, Germany). Each biological sample was sonicated in an ultrasonication bath three times for 4 min, with 2 min between bursts and left in ice for 1 h. After lysis, each sample was centrifuged at 16,000 x g for 10 min, at 4°C, and the supernatant was collected. The protein concentration was determined using the Quick Start TM Bradford Protein Assay and 500 μg of each sample was analysed with preparative 12% T SDS-PAGE under reducing conditions. For protein identification, preparative SDS-PAGE gel lanes corresponding to each biological sample were cut into 24 parallel slices, which were triturated, washed with water and acetonitrile, S-alkylated with 55 mM iodoacetamide in 100 mM NH_4_HCO_3_, washed again, and finally digested with trypsin (12.5 ng/μL in 50 mM NH_4_HCO_3_, 5 mM CaCl_2_). The resulting peptide mixtures were desalted/concentrated using ZipTip C18 (Merck), vacuum-dried in a Savant roto-evaporator (Thermo Fisher Scientific) and finally reconstituted in 0.1% v/v formic acid prior to MS analysis. A unique technical replicate was accomplished for each biological sample.

Peptide mixtures were analyzed by means of a nanoLC-ESI-Q-Orbitrap-MS/MS platform consisting of an UltiMate 3000 HPLC RSLC nano-chromatography system (Thermo Fisher Scientific, Bremen, Germany) linked to a Q-ExactivePlus mass spectrometer through a Nanoflex ion source (Thermo Fisher Scientific, Bremen, Germany). Peptides were loaded onto an Acclaim PepMapTM RSLC C18 column (150 mm × 75 μm ID, 2 μm particles, 100 Å pore size) (Thermo Fisher Scientific), and eluted with a gradient of solvent B (19.92/80/0.08 v/v/v water/acetonitrile/formic acid) in solvent A (99.9/0.1 v/v water/formic acid), at a flow rate of 300 nL/min. The gradient of solvent B started at 3%, increased to 40% over 50 min, increased to 80% over 5 min, remained at 80% for 4 min, and finally returned to 3% in 1 min, with a column equilibrating step of 20 min before the subsequent chromatographic run. The mass spectrometer operated in data-dependent mode, using a full scan range (*m/z* 375–1500, resolution of 70,000 @200 *m/z*), followed by MS/MS scans of the 10 most abundant ions. MS/MS spectra were acquired in a dynamic scan *m/z* range, using a normalized collision energy of 28%, an automatic gain control target of 100,000, a maximum ion target of 120 ms, and a resolution of 17,500 @200 *m/z*. The dynamic exclusion value was set to 30 s.

### 2.9 Bioinformatic analysis for protein identification and functional assignment

For protein identification, all raw mass data files associated with protein digests of slices from the same biological sample were merged in a unique file that was subjected to analysis by Proteome Discoverer v. 2.4 software (Thermo Fisher Scientific), enabling database search with Mascot algorithm v. 2.4.2 (Matrix Science) according to a shotgun proteomic approach (Perkins et al., 1999; Renzone et al., 2015). Database searching was performed using a *B. oleracea* L. protein sequence database downloaded from UniProtKB (121,712 entries, 01/2024), with the following criteria: carbamidomethylation at cysteine as fixed modification, and oxidation at methionine, pyroglutamate formation at N-terminal glutamine and phosphorylation at serine/threonine/tyrosine as variable modifications. Parent peptide mass tolerance was set to ±10 ppm and to ±0.05 Da for MS/MS fragments. Trypsin was set as the proteolytic enzyme, and the maximum number of missed cleavages was limited to 2. Protein candidates were considered confidently identified only when the following criteria were satisfied: i) protein and peptide false discovery rate (FDR) confidence: high (i.e., >99%); ii) peptide Mascot score >25; iii) peptide spectrum matches (PSMs): unambiguous; iv) rank of the peptide match (peptide rank): 1 (namely, the best match); v) normalized score difference between the selected PSM and the highest-scoring PSM for that spectrum (Delta CN): 0. The proteomic data were deposited in the ProteomeXchange Consortium via the PRIDE partner repository (Perez-Riverol et al., 2022) with the dataset identifier PXD063392.

*B. oleracea* PDNV proteins were functional categorized using Mercator pipeline (Schwacke et al., 2019) for automated sequence annotation, selecting TAIR 10, SwissProt-UniProtKB plant proteins, KOG clusters and InterPro scan, with a cut-off value of 80, as previously reported (Salzano et al., 2019). Protein association network analysis and functional annotations were obtained with STRING software v. 12 (Szklarczyk et al., 2023) using the proper organism database. Result output integrated Gene Ontology (GO) aspects, such as Molecular Function (MF), Cellular Component (CC), and Biological Process (BP) (The Gene Ontology Consortium, 2017), and Reactome (Milacic et al., 2024), Kyoto Encyclopedia of Genes and Genomes (KEGG) (Kanehisa et al., 2017), and BioCyc (Caspi et al., 2020) annotations.

### 2.10 Lipid extraction and LC-HR-MS/MS analysis

Pooled samples of anion exchange-FPLC purified *B. oleracea* PDNVs (0.1 mL) were extracted according to Folch method (Eggers and Schwudke, 2016) with slight modifications; each fraction was mixed with chloroform: methanol (2/1, v/v, 2 mL), and acidification was achieved upon the addition of 0.05 mL of 0.1 N HCl. The samples were gently mixed and then centrifuged at 18,000 x g, for 20 min, 4°C. The supernatant was collected, dried under nitrogen stream and finally dissolved in 80% v/v isopropanol for further LC-HR-MS/MS analysis. An Exploris 120 quadrupole Orbitrap high-resolution mass spectrometer was interfaced with a Vanquish Core liquid chromatographic system (Thermo Fisher Scientific). The mobile phases consisted of 5 mM ammonium formate with 0.1% v/v formic acid in water: acetonitrile 60/40 v/v (solvent A) and 5 mM ammonium formate with 0.1% v/v formic acid in isopropanol: acetonitrile 90/10 v/v (solvent B); the flow rate was 0.2 mL/min. A reversed phase C18 core shell column (Kinetex C18 PS, 100 × 2.1, 2.6 µm, Phenomenex, Torrance, CA) was used with the following solvent B gradient (min/%B): (0/20), (0.5/20), (3/50), (10/70), (18/97), (22/97) at 50°C, with an equilibration stage of 8 min. For untargeted analysis, sample runs were acquired in polarity switching mode, scanning the ions in full scan mode in the *m/z* range 120–1,500. Heated electrospray interface (H-ESI) static spray voltage was set at −3.2 kV in negative ions and 3.5 kV for positive ions; ion transfer tube and vaporizer temperatures were set at 300°C and 320°C, respectively; sheath gas and auxiliary gas flow values were set 50 and 10 arbitrary units, respectively. The analyzer resolution was set at 60,000 (FWHM at *m/z* 200) and data acquired with a normalized all-gain control (AGC) target of 100%. Quality control samples were obtained by mixing technical replicates of purified PDNV samples and analyzed within the batch. Full scan acquisition mode was further completed with data-dependent scanning experiments (ddMS^2^) covering differential scan ranges (*m/z* 120–450, *m/z* 440–750, *m/z* 740–1,050, *m/z* 1,040–1,500, resolution 60000, FWHM at *m/z* 200) and working alternatively in positive or negative ion mode for maximzing unknown compounds identification. For ddMS^2^ experiments, an isolation window of *m/z* 1.1 and normalized collision energies at 20%, 35% and 60% were used. The intensity threshold was fixed at 45,000 area counts, the dynamic exclusion was set by considering a time window of 2.5 s and a mass tolerance between 5 ppm. A targeted mass exclusion list was obtained in the full scan mode by injecting blank procedural and blank solvent samples. EASY-IC with fluoranthene was used in ddMS^2^ positive (*m/z* 202.0777 [M]^+^) and in ddMS^2^ negative ion mode (*m/z* 202.0788 [M]^-^) to optimize mass accuracy. Profile data were analyzed using Xcalibur 4.5 (Thermo Fisher Scientific, Waltham, MA), while fragmentation spectra were recorded by using Free Style software v. 1.8 (Thermo Fisher Scientific).

### 2.11 Metabolite extraction and LC-HR-MS/MS analysis

Polar and non-polar metabolites were extracted from anion exchange-FPLC purified *B. oleracea* PDNVs according to Barouh and colleagues (Barouh et al., 2024) with slight modifications; 1.2 mL of Folch mixture (chloroform/methanol/water, 8:4:3 v/v) and 80 µL of 0.1 N HCl were added to 100 µL of PDNV samples and vortexed for 5 min. Successively, 300 µL of Folch mixture and 25 µL of 0.735% w/v NaCl were added and the mixture vortexed for 5 min, and then stirred at room temperature for 1 h. Finally, the samples were centrifuged at 800 x g for 5 min, and the corresponding hydroalcoholic fraction phase was dried in a centrifuge evaporator (Savant, Thermo Fisher), resuspended in 100 µL of methanol/water (70:30, v/v) and used for polar and non-polar phytometabolite analysis. The chromatographic separation encompassed the use of a biphenyl core shell column (Kinetex biphenyl, 100 × 2.1, 2.6 µm, Phenomenex, Torrance, CA) thermostated at 35°C, and eluted at flow rate of 0.3 mL/min. The elution solvents were 0.1% v/v formic acid in water (solvent A) and acetonitrile (solvent B), which were used with the following gradient (min/%B): (0/5), (0.5/5), (1.5/15), (10/40), (15/70), (16/95). Sample runs were acquired in positive and negative polarity switching mode, scanning the ions in full scan mode in the *m/z* range 120–1,500. The conditions for HRMS were set as follows: heated electrospray interface (H-ESI) static spray voltage was −3.2 kV in negative ions and 3.5 kV for positive ions; ion transfer tube and vaporizer temperatures were set at 300°C and 320°C, respectively; sheath gas and auxiliary gas flow values were set 50 and 10 arbitrary units, respectively. The analyser resolution was set at 60,000 (FWHM at *m/z* 200) and data were acquired in profile mode with a normalized AGC target of 100%. Two technical replicates were performed for each experiment.

### 2.12 Untargeted bioinformatic analysis for lipid and metabolite identification

The full scan and ddMS^2^ raw files were imported in Compound Discoverer 3.3 (Thermo Fisher Scientific, San José, CA). An untargeted workflow based on the identification of phytochemicals, primary and secondary polar and lipid apolar metabolites was used. The software grouped similar compounds according to their chemical features including retention time, diagnostic ions, molecular mass, and formula. Identification was achieved through exact masses, formulas and fragmentation spectra against MS fragmentation patterns available in databases such as mzCloud (https://www.mzcloud.org), ChemSpider (https://www.chemspider.com), Phenol-Explorer (http://phenol-explorer.eu), Natural Products Atlas (https://www.npatlas.org), LipidMaps (https://lipidmaps.org), and KEGG (https://www.genome.jp/kegg). An internal mass list was generated starting with flavonoids structure database for polar and non-polar metabolites as well as LipidMaps structure database for lipid apolar compounds (Fukushima et al., 2022). Annotated compounds were further screened for putative identification through the analysis of available standards, matching with diagnostic ions reported in publicly available databases and in previous studies (Claassen et al., 2019) Along with LC-HR-MS/MS *m/z* exclusion list and noise removal, multivariate filtering procedure was applied to background and solvent removal, presence of normalized area, fragmentation spectra and molecular formula. Calibration curves were constructed through the standard addition technique and the concentration of target compounds was reported in ng/mL of the PDNV fraction. The original contributions presented in the study are included in the article and in the supplementary material. Furthermore, datasets and the raw data supporting the conclusions of this article are available by the authors on request.

### 2.13 Total RNA extraction

For the isolation of total RNA from *B. oleracea* PDNVs, 1 mL of NucleoZOL reagent (MACHEREY-NAGEL GmbH & Co. KG) was mixed with 400 µL of anion exchange-FPLC purified PDNV samples. After the addition of 400 µL of RNase-free water, samples were vigorously vortexed and centrifuged at room temperature at 12,000 x g for 10 min. The aqueous phase containing total RNA was precipitated by adding an equal volume of isopropanol. The RNA pellet was washed twice with 75% v/v ethanol and resuspended in 25 µL of RNase-free water. Total RNA concentration was quantified using a NanoDrop spectrophotometer. Extraction was performed on three biological replicates of FPLC purified PDNVs and these samples were subsequently used for miRNA sequencing.

### 2.14 SmallRNA sequencing

RNA quality was further assessed by Qubit 4.0 and TapeStation 4200 (Agilent Technologies) systems. Indexed libraries were prepared from 5 μL of the isolated RNA using QIAseq^®^ miRNA UDI Library Kit (Qiagen), according to the manufacturer’s instructions. Libraries were quantified using the TapeStation 4200 instrument (Agilent Technologies) and a Qubit fluorometer (Invitrogen Co.); then, they were pooled such that each index-tagged sample was present in equimolar amounts. The pooled samples were subject to cluster generation and sequencing using an Illumina Novaseq6000 System (Illumina) in a 1 × 75 single-end format. Sequence data were deposited at the RSA database with the dataset identifier PRJNA1259063 (https://www.ncbi.nlm.nih.gov/sra/PRJNA1259063).

### 2.15 Bioinformatic analysis for microRNA identification

The quality of raw data from smallRNA sequencing was evaluated using FastQC (v0.12.1) available at http://www.bioinformatics.babraham.ac.uk/projects/fastqc (Andrews, 2010). The analysis in FastQC provided a report containing statistics, graphs and tables, which allowed to spot potential problems originated either in the sequencer or in the starting library material. Thus, the sequenced reads were checked for quality and for the presence of sequencing adapters. The bioinformatic tool sRNAbench (Aparicio-Puerta et al., 2019) was used to perform both low-quality reads and adapter sequences removal; the latter processes were accomplished using the Qiagen QIAseq miRNA protocol option. sRNAbench was used to obtain the expression profiles of microRNAs. Expression values were obtained by the option library mode in which reads were mapped directly against the reference sequences. The reference database was PmiREN2.0 (Guo et al., 2020), selecting the following related species: *B. juncea* (Bju), *B. napus* (Bna), *B. nigra* (Bni), *B. oleracea* (Bol), and *B. rapa* (Bra). The expression of mature miRNAs in each library was normalized to Counts Per Million reads (CPM) as follows: CPM = (miRNA read counts/total mapped read counts per library) × 1,000,000. psRNAtarget (Dai et al., 2018) was applied to identify human mRNA putative targets of the identified miRNAs, choosing the *Homo sapiens* transcriptome as reference. To obtain an overview of the biological processes, molecular functions and cellular components modulated by all the predicted targets for the clusters of miRNAs expressed in each sample, a Gene Ontology (GO) enrichment analysis was performed by Metascape (Zhou et al., 2019).

### 2.16 Skin cell cultures

Immortalized Human Keratinocytes (HaCaT), bought from Addexbio Technologies (San Diego, CA, USA), were maintained in Dulbecco’s Modified Eagle Medium (DMEM; Sigma Aldrich, St. Louis, MO, USA) supplemented with 10% v/v fetal bovine serum (FBS) (Sigma Aldrich, St. Louis, MO, USA), in 95% v/v air, 5% v/v CO_2_ and humidified atmosphere, at 37°C. Murine RAW 264.7 macrophages were obtained from the European Collection of Cell Cultures (ECACC; Health Protection Agency Culture Collection, Porton Down, UK). The cells were cultured in DMEM (Gibco) supplemented with 2 mM L-glutamine, 4.5 g/L glucose, 1 mM sodium pyruvate, 1.5 g/L sodium bicarbonate, and 10% v/v FBS, under a humidified atmosphere containing 5% v/v CO_2_ at 37°C. The *in vitro* assays were performed at Arterra Bioscience, Naples, Italy. For all the biological assays, three technical replicates were performed for each of the two biological replicates.

### 2.17 Treatment of skin cell lines with PDNVs

HaCaT and RAW 264.7 (murine macrophages) cell lines were cultured in DMEM supplemented with 10% v/v FBS, 1% w/v penicillin, and 1% w/v streptomycin, and maintained at 37°C with 5% v/v CO_2_. All experiments were carried out when the cell cultures reached 70%–80% confluence. Each cell type was uniformly treated with fresh media containing either 1 × 10^7^ or 1 × 10^6^ particles/mL of anion exchange-FPLC purified *B. oleracea* PDNVs, or mock-treated with TGF-β or dexamethasone as positive control.

### 2.18 Cytotoxicity assay

Cytotoxicity assays were performed using 3-(4,5-dimethylthiazolyl)-2,5-diphenyltetrazolium bromide (MTT). Cells were cultured in 96-well plates using DMEM supplemented with 10% v/v FBS and incubated for approximately 8 h. After treatment with purified PDNVs at concentrations of 1 × 10^8^, 1 × 10^7^, or 1 × 10^6^ particles/mL, for 48 h, cells were washed with PBS and incubated with 100 μL/well of reaction buffer containing 10 mM Hepes, 1.3 mM CaCl_2_, 1 mM MgSO_4_, 5 mM glucose, and 0.5 mg/mL MTT substrate in PBS, pH 7.4. After 3 h of incubation at 37°C with 5% v/v CO_2_, 100 μL of solubilizing solution (10% Triton X-100, 0.1 N HCl in absolute isopropanol) was added to each well. The colorimetric reaction was allowed to proceed for 16 h, and the absorbance was measured at 595 nm using a Victor3 Nivo plate reader (PerkinElmer Inc., Singapore).

### 2.19 Scratch assay

HaCaT cells were seeded in 6-well plates at a density of 8× 10^5^ cells per well. After 6 h of incubation, a scratch was made through the cell monolayer of each well, using a pipette tip to generate a uniform cell-free area. The detached cells were removed by washing with PBS. Fresh medium with 0.5% v/v FBS, containing or not PDNVs (1 × 10^8^, 1 × 10^7^, or 1 × 10^6^ particles/mL) was then added, and cells were incubated for additional 7 h. TGF-β (2.5 ng/mL) was used as a positive control. In order to evaluate cell migration, the remaining cell-free areas were measured at 0 and 16 h after treatment for each condition. The quantification was performed using ImageJ software.

### 2.20 Analysis of gene expression in scratch-wounded HaCaT cells

Total RNA was extracted from scratch-wounded HaCaT cells, which were treated with purified *B. oleracea* PDNVs as reported above, or untreated. Each group was processed in triplicate, categorized as one set, with two independent sets prepared for semi-quantitative RT-PCR analysis. Total RNA was extracted using the GenElute™ Total RNA Purification Kit (Sigma-Aldrich, Saint Louis, MI, USA) and then treated with DNase I (Thermo Scientific, Waltham, MA, USA) at 37°C, for 30 min, to remove contaminating genomic DNA. A total of 500 ng of RNA was retro-transcribed using the Reverse Transcriptase enzyme (Thermo Scientific, Waltham, MA, USA). Semi-quantitative RT-PCR was performed with universal 18S primer/competimer pairs (Invitrogen, Thermo Scientific, Waltham, MA, USA), as internal standards. PCR products were separated on a 1.5% w/v agarose gel, and images were captured using the iBright imaging system (Invitrogen, Thermo Scientific, Waltham, MA, USA). The sequences of the primers used for amplification were the following:

**Table:** 

Primers	Sequence
EGFR Fw	ATGTCCTCATTGCCCTCA
EGFR Rv	CACATCCATCTGGTACGT
Ki-67 Fw	CTA​GAA​ATC​GAA​CAC​CAG​C
Ki-67 Rv	GGA​TTT​CTG​AAC​CTG​ACT​C
Coll IV Fw	TGTGACCAGGCATAGTCA
Coll IV Rv	GAGCCAAAAGCTGTAAGC
LAM5-γ Fw	ATC​AAC​AGG​TGA​GCT​ATG​G
LAM5-γ Rv	CAA​TCT​CCT​GTG​TCT​GGA​T

### 2.21 Nitric oxide assay in LPS-stimulated RAW 264.7 murine macrophages

Nitric oxide (NO) levels were quantified in RAW 264.7 murine macrophages, seeded in 96-well plates at a density of 1.5 × 10^5^ cells per well and incubated for 24 h. Cells were pre-treated with different concentrations of purified *B. oleracea* PDNVs, with 10 μM N-tosyl-L-phenylalanine methyl ketone (TPCK) used as a positive control, for 2 h, followed by stimulation with 2 μg/mL LPS for 18 h. Nitric oxide, measured as nitrite, was determined using the Griess reagent, a solution of N-(1-naphthyl)ethylenediamine and sulfanilic acid (Invitrogen, Thermo Scientific, Waltham, MA, USA). After 30 min, the absorbance was read at 540 nm with a multiwell plate reader Victor3 Nivo plate reader (PerkinElmer Inc., Singapore).

### 2.22 Analysis of inflammatory genes expression in LPS-stimulated HaCaT cells

HaCaT cells (1.5 × 10^5^ cells per well) were seeded in 6-well plates and cultured for 8 h. Subsequently, cells were treated overnight with anion exchange-FPLC purified *B. oleracea* PDNVs or 10 μM dexamethasone (used as a positive control) under the conditions described above. After treatment, inflammatory stress was induced by adding 5 μg/mL LPS. Total RNA extraction was carried out 6 h after the induction. Each group was processed in triplicate, categorized as one set, with two independent sets prepared for semi-quantitative RT-PCR analysis. Total RNA was extracted using the GenElute™ Total RNA Purification Kit (Sigma-Aldrich, Saint Louis, MI, USA) and subsequently treated with DNase I (Thermo Scientific, Waltham, MA, USA) at 37°C, for 30 min, to remove contaminating genomic DNA. A total of 500 ng of RNA was retro-transcribed using the Reverse Transcriptase enzyme (Thermo Scientific, Waltham, MA, USA). Semi-quantitative RT-PCR was performed with universal 18S primer/competimer pairs (Invitrogen, Thermo Scientific, Waltham, MA, USA), as internal standards. The PCR products were separated on a 1.5% w/v agarose gel, and images were captured using the iBright imaging system (Invitrogen, Thermo Scientific, Waltham, MA, USA). The sequences of the primers used for amplification were the following:

**Table:** 

Primers	Sequence
IL-1β Fw	ATG​GCA​GAA​GTA​CCT​GAG​CT
IL-1β Rv	AAG​GAC​ATG​GAG​AAC​ACC​AC
TLR2 Fw	CTGGGCAGTCTTGAACAT
TLR2 Rv	CAT​CTT​TTC​TGG​CCA​CTG​ACA

### 2.23 Statistical analysis

Statistical analyses were performed with the Graphpad Prism 9.5 software. Data are presented as the mean ±SEM from at least three independent experiments. The statistical differences between the groups were determined using a one-way analysis of variance (ANOVA), followed by Tukey’s *post hoc* test for pairwise comparisons. A *p*-value < 0.05 (two-tailed) was considered statistically significant.

## 3 Results

### 3.1 Purification of PDNVs from *B. oleracea* seedlings by anion exchange-FPLC

**FIGURE 1:**
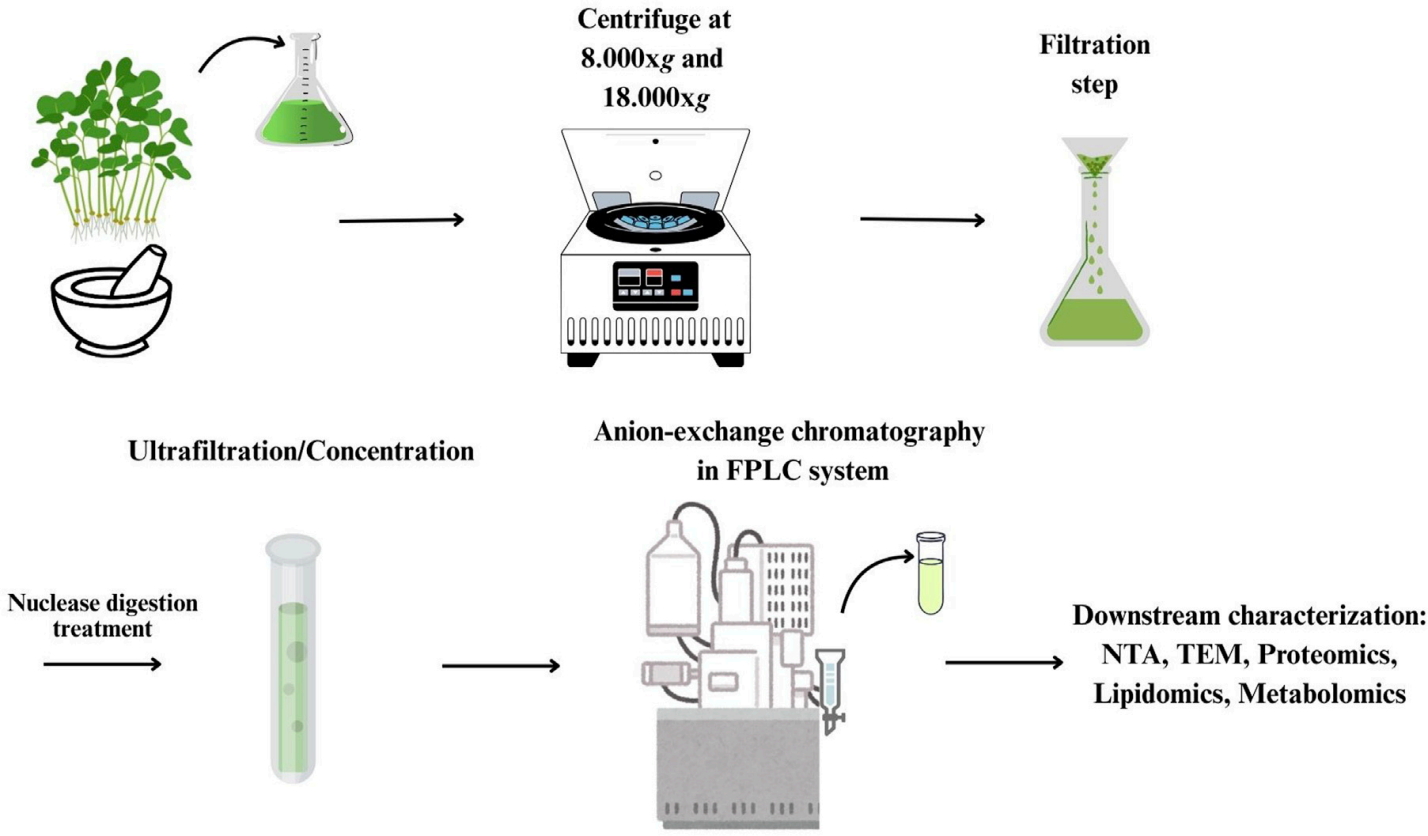
Workflow of the purification procedure for the isolation of *B. oleracea* PDNVs.

**FIGURE 2:**
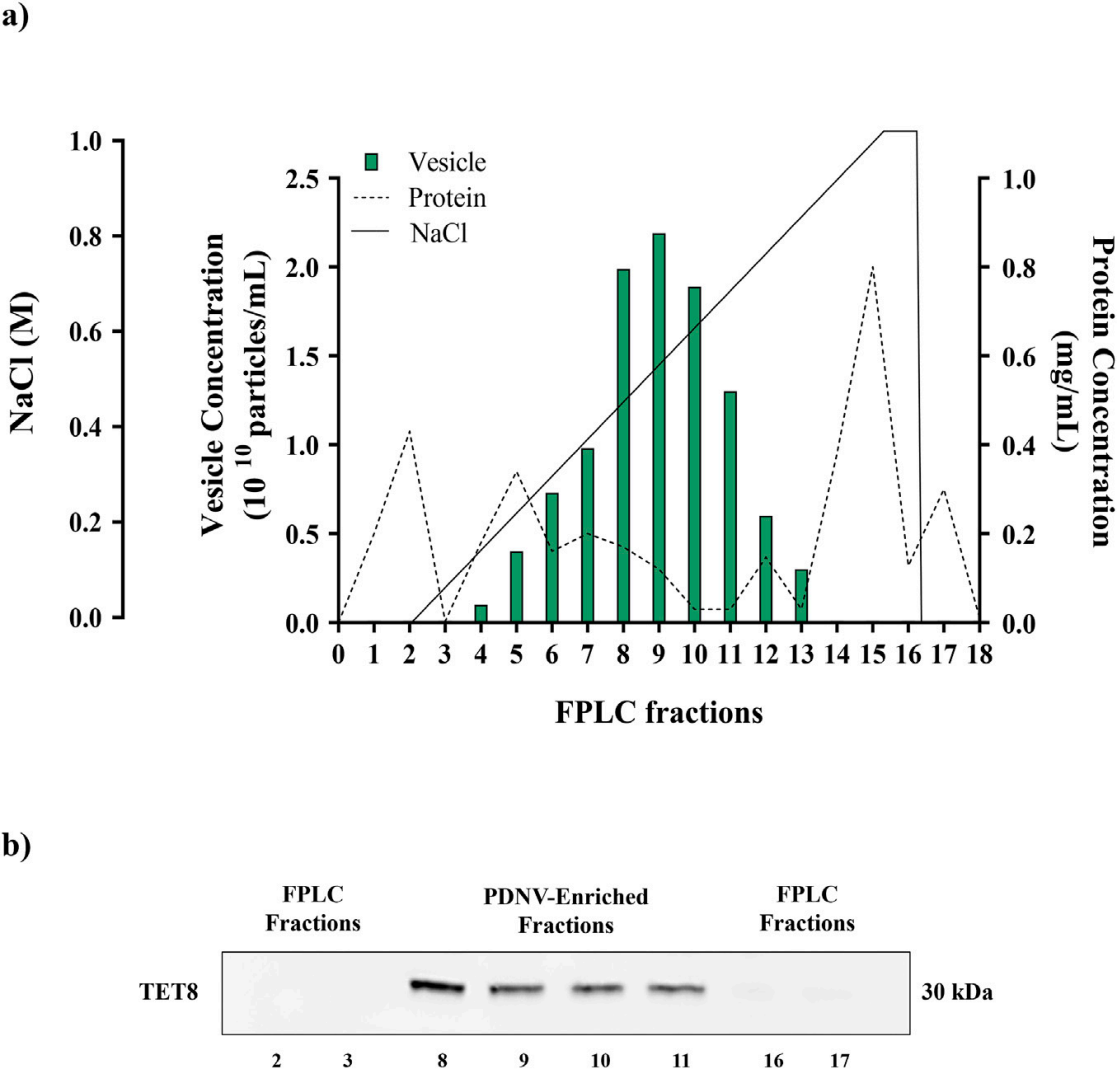
FPLC purification of *B. oleracea* PDNVs. **(a)** FPLC chromatogram showing the elution of proteins and PDNVs. The ultrafiltered sample was loaded on a FPLC apparatus equipped with a CIMmultus™ EV monolith column and eluted with a 0–1 M NaCL gradient under conditions described in Materials and Methods. **(b)** Western blot of FPLC fractions. Samples of FPLC fractions (2–3; 8–11; 16–17) were blotted and incubated with the anti-TET8 antibody, under conditions reported in Materials and Methods. A representative experiment is reported.

After centrifugation to remove large cellular debris, the homogenate of the aerial part of *B. oleracea* L. seedlings was subjected to sequential filtration and then to ultrafiltration (cut off-100 kDa) to remove low-mass contaminants. The resulting retentate was subjected to diafiltration to allow buffer exchange for nuclease treatment, in order to remove high molecular weight nucleic acid/protein aggregates. After nuclease treatment, the sample was diafiltered against an anion exchange buffer and subjected to anion exchange-chromatography in a FPLC system (Figure 1). Elution was performed by applying a linear 0–1 M NaCl gradient in 35 min. In the collected fractions, protein and PDNV concentrations were estimated by Bradford assay and NTA analysis, respectively. The elution chromatogram, reported in Figure 2a, showed that PDNVs were most concentrated in fractions 8–11, while most contaminant proteins eluted at lower or higher NaCl concentrations. To confirm the presence of PDNVs, a Western blot analysis was performed using an antibody against TET8 protein, a widely accepted PDNV marker. The analysis revealed the presence of a strong TET8 signal in fractions 8 to 11, where PDNVs were concentrated, while it was markedly reduced or completely absent in fractions containing most of contaminant proteins, such as fraction 2,3 and 16,17 showed in the blotting (Figure 2b).

**FIGURE 3:**
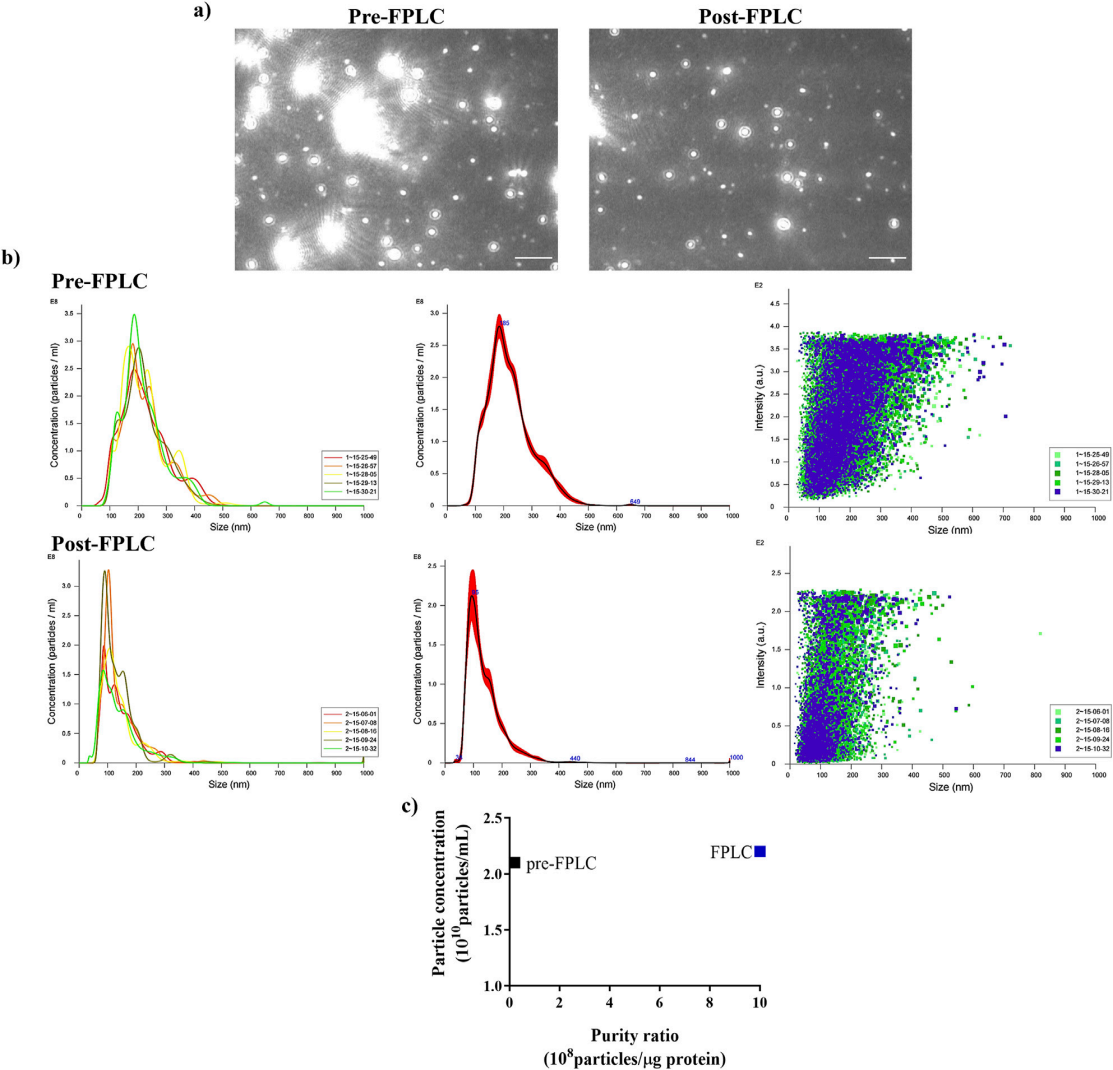
Nanoparticle Tracking Analysis (NTA) of ultrafiltered (pre-FPLC) *B. oleracea* sample and of FPLC-purified pooled 8–11 fractions (post-FPLC). **(a)** Nanovesicle images from NTA analysis. **(b)** NTA-calculated distribution of PDNVs sizes. **(c)** Purity index, calculated as n° of particles/μg protein ratio. A representative experiment is reported. Scale bar = 1 μm.

To evaluate the effectiveness of FPLC purification, NTA analysis of pooled 8–11 fractions (post-FPLC) was performed and compared to that of the ultrafiltered sample (pre-FPLC). The NTA images reported in Figure 3a, showed that in the pre-FPLC sample numerous bright spots of higher molecular mass as compared to the rest of vesicle population were present, whose number appeared significantly reduced in the post-FPLC sample. Therefore, the analysis demonstrated that the post-FPLC sample contained a more homogeneous PDNV population than that of the pre-FPLC sample, a fact due to a significan reduction in the content of large molecular mass contaminants, such as protein aggregates or nucleosomes (Gagnon et al., 2020), brought about by anion exchange chromatography.

**FIGURE 4:**
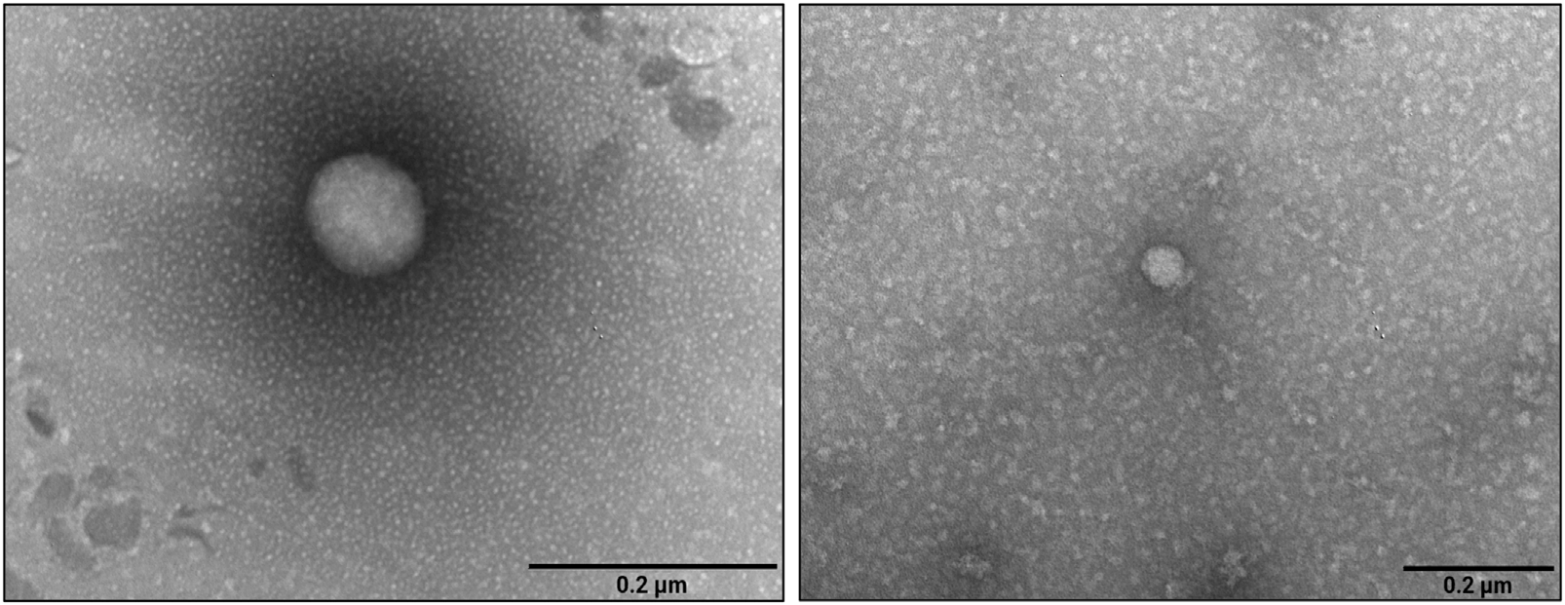
Transmission electron microscopy images of FPLC-purified *B. oleracea* PDNVs. Samples were negatively stained, air-dried and analyzed as reported in Materials and Methods. Scale bar = 200 nm.

NTA analysis of the pre-FPLC sample (Figure 3b) showed that PDNVs had a Gaussian size distribution with D10 (10% population) of 130.6 nm, D90 of 335.5 nm and a mean dimension of 221.9 nm. In the post-FPLC sample, PDNVs had D10 and D90 of 79.2 and 215.7 nm, respectively, and a mean dimension of 137.8 nm, as indicated by the reduced area of the peak. The concentration of PDNVs was 2.17–2.38 × 10^10^ particles mL^-1^ in the pre-FPLC sample and 1.99–2.2 × 10^10^ particles mL^-1^ in the post-FPLC sample, while the purity index, calculated as n° of particles μg^-1^ protein, was 1.0 × 10^9^ in the post-FPLC sample, as compared to 0.1 × 10^8^ of pre-FPLC one (Figure 3c). Overall, these results indicated that anion exchange FPLC removed about 90% of contaminants, while almost unaffecting the total number of PDNVs; this finding proved the effectiveness of the chromatographic purification. The FPLC-purified PDNVs were then analyzed by TEM. The images reported in Figure 4 showed that the PDNVs exhibited a well-defined sperical shape, with a clear boundary between the lipid bilayer membrane and the surrounding medium, with a population size ranging from 30 (panel a) to 200 nm (panel b), and with an average diameter of approximately 120 nm. The size distribution observed by TEM well correlated to that obtained by NTA analysis, confirming the consistency of the isolation method and also demonstrating that the FPLC purification preserved the phisiological morphology of the extracellular vesicles.

### 3.2 Proteomic analysis

**FIGURE 5:**
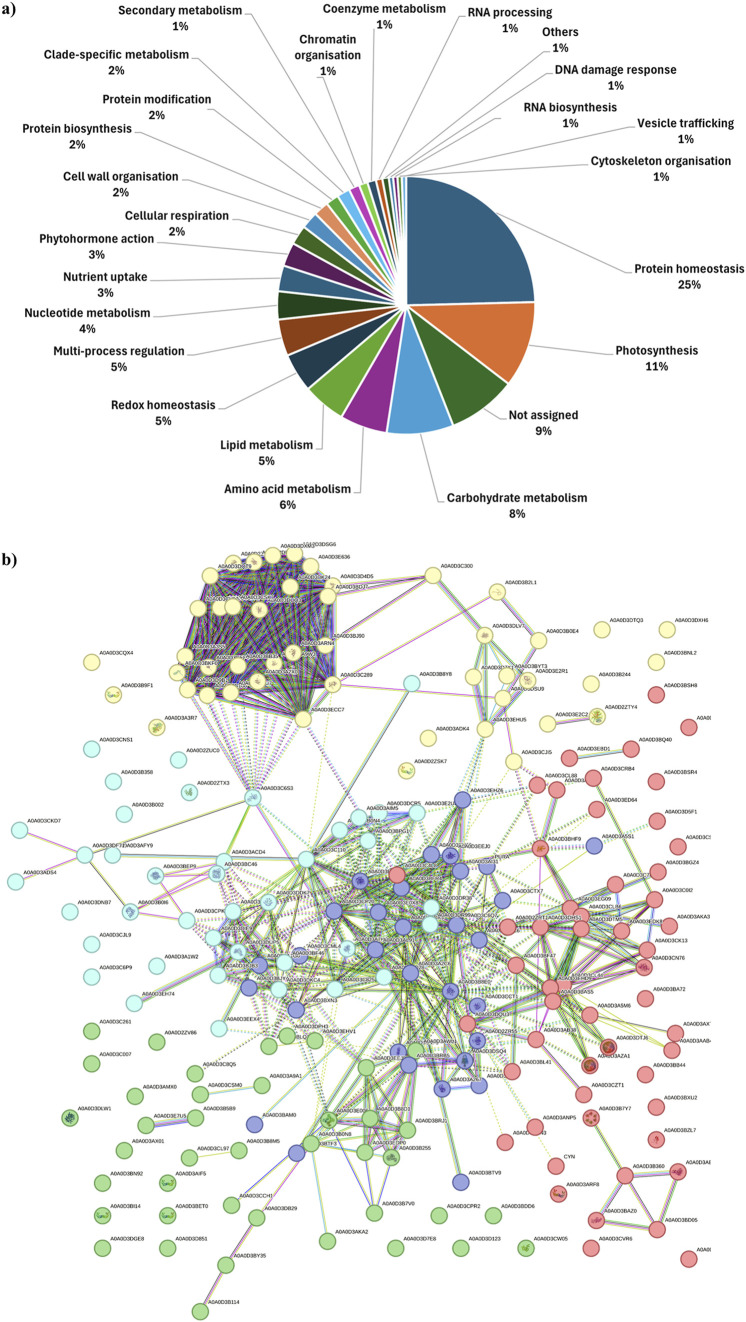
Functional classification and association interaction network of proteins identified in FPLC-purified *B. oleracea* PDNVs. **(a)** Assigned proteins were categorized for their function with Mercator software as reported in Materials and Methods. **(b)** The protein association network was built using the proteins reported in Table S1, which were aligned to the *B. oleracea* sequence database within the STRING suite allowing to build up 1047 medium-confidence interactions (0.4). Protein functional annotation was automatically performed by the software based on information described in Materials and Methods. Recognized functional clusters are reported in different colors and include proteins associated with: proteasomal protein processes (yellow, 47 proteins); protein folding (red, 53 proteins); apoplast/secretory vesicles (green, 41 proteins); metabolic processes (blue; 40 proteins); inter-intracellular communication (purple, 31 proteins). Red knots. A0A0D2ZRT1 and A0A0D3BF47: CN hydrolase domain-containing proteins; A0A0D3A828: 60S ribosomal protein L27; A0A0D3DYA3, A0A0D3AAB4, A0A0D3BGZ4, A0A0D3BQ40, A0A0D3BSR4, A0A0D3C0I2, A0A0D3C4L8, A0A0D3C7K2, A0A0D3CK13, A0A0D3CZT1, A0A0D3D5F1, A0A0D3EBD1, A0A0D3ED64, A0A0D3EDK8, A0A0D3CN76, A0A0D3AKA3 and A0A0D3AXT8: uncharacterized proteins; A0A0D3AB38: member of the glycosyl hydrolase 31 family; A0A0D3AE77 and A0A0D3BAZ0: members of the heat shock protein 70 family; A0A0D3ANP5: urease; A0A0D3ARF8, 14-3-3 protein isoforms; A0A0D3AZA1 and A0A0D3DTJ6: ferritin isoforms; A0A0D3B360: H-ATPase_c domain-containing protein; A0A0D3B7Y7, member of the small heat shock protein (HSP20) family; A0A0D3BA72: DOMON domain-containing protein; A0A0D3CL44, A0A0D3BAS5 and A0A0D3EHD9: thioredoxin domain-containing proteins; A0A0D3BB44, A0A0D3BD05, A0A0D3BSH8 and A0A0D3CSF1: members of the chaperonin (HSP60) family; A0A0D3BDN3: proline iminopeptidase; A0A0D3BHF9: universal ribosomal protein uS8; A0A0D3BXU2: pectate lyase; A0A0D3BZL7: ribulose bisphosphate carboxylase small chain; A0A0D3A5M6 and A0A0D3BL41: aminotran1-2 domain-containing proteins; A0A0D3CL88: SGNH_hydro domain-containing protein; A0A0D3CLB6 and A0A0D3DTM5: Fe-Mn superoxide dismutase isoforms; A0A0D3CRB4: phospholipase D; A0A0D3CVR6: Nudix hydrolase domain-containing protein; A0A0D3DHS1: catalase; A0A0D3DQU3: protein disulfide-isomerase; A0A0D3EG09: RRM domain-containing protein; A0A0D3EG13: cysteine proteinase inhibitor; CYN: cyanate hydratase. Yellow knots. A0A0D2ZSK7, A0A0D3B9F1 and A0A0D3E2C2: 14-3-3 protein isoforms; A0A0D2ZTY4, glutathione S-transferase; A0A0D3D7X1 and A0A0D3E2R1: ferredoxin-NADP reductase, chloroplastic; A0A0D3A3R7: seed storage protein; A0A0D3B2L1: core component of nucleosome; A0A0D3C300: E1 ubiquitin activating enzyme; A0A0D3B244: germin-like protein; A0A0D3CQX4: glutamine-dependent NAD(+) synthetase; A0A0D3DK24: proteasome endopeptidase complex component; A0A0D3DLV7: cysteine synthase; A0A0D3ECC7: AAA-ATPase subunit; A0A0D3ADK4: BED-type domain-containing protein; A0A0D3DXH6: purple acid phosphatase; A0A0D3DSU9: histone H4; A0A0D3DTQ3: calmodulin; A0A0D2ZV84, A0A0D3C289, A0A0D3ARN4, A0A0D3CVU7, A0A0D3DSG6, A0A0D3E636 and A0A0D3D4D5: proteasome subunit alpha type isoforms; A0A0D3A2X0, A0A0D3BDJ7, A0A0D3CH47, A0A0D3DPF1, A0A0D3DQT9, A0A0D3DUQ3, A0A0D3CV17 and A0A0D3DXM3: proteasome subunit beta isoforms; A0A0D3A3S5: 26S proteasome non-ATPase regulatory subunit 2 homolog; A0A0D3A9W3: proteasome subunit beta belonging to the peptidase T1B family; A0A0D3CJI5: TCTP-domain containing protein; A0A0D3BNL2: peptidase_S9 protein; A0A0D3BKF0, A0A0D3D106, A0A0D3D9B1 and A0A0D3CEF4: PCI domain-containing proteins; A0A0D3B0E4, A0A0D3BB35, A0A0D3BYT3, A0A0D3CSK7 and A0A0D3EHU5: uncharacterized proteins; A0A0D3BJ90: VWFA domain-containing protein. Green knots. A0A0D2ZV86: purple acid phosphatase; A0A0D3CL97: clathrin heavy chain; A0A0D3C8Q5: epimerase; A0A0D3B255, A0A0D3E006 and A0A0D3EDP0: sucrose synthase isoforms; A0A0D3BLQ1: aminopeptidase; A0A0D3B8M5: F-box domain-containing protein; A0A0D3AKA2, A0A0D3B5B9, A0A0D3B7V0, A0A0D3AX0, A0A0D3DPH13, A0A0D3E7U5, A0A0D3D123, A0A0D3C007, A0A0D3CCH1, A0A0D3CPR2: uncharacterized proteins; A0A0D3AWL1: alpha-1,4 glucan phosphorylase; A0A0D3B114 and A0A0D3DB29: alpha-mannosidase isoforms; A0A0D3AIF5, A0A0D3BET0, A0A0D3BI14 and A0A0D3CW05: 14-3-3 domain-containing proteins; A0A0D3CSM0: peptidase_S9 protein; A0A0D3BDD6: FAS1 domain-containing protein; A0A0D3BY35: member of the glycosyl hydrolase 2 family; A0A0D3B0N8: member of the glycosyl hydrolase 31 family; A0A0D3DLW1: member of the glycosyl hydrolase 1 family; A0A0D3C261: PITH domain-containing protein; A0A0D3D7E8: MBD domain-containing protein; A0A0D3D851: member of the peptidase S8 family; A0A0D3EHV1: member of the peptidase M18 family; A0A0D3DGE8: phytocyanin domain-containing protein; A0A0D3EEJ9: phosphoacetylglucosamine mutase; A0A0D3B8D1: isoamylase 3, chloroplastic; A0A0D3BRJ1: 1,4-alpha-glucan-branching enzyme 2-1, chloroplastic/amyloplastic; A0A0D3BN92: member of the chaperonin (HSP60) family; A0A0D3AMX0: CPSF_A domain-containing protein; A0A0D3A9A1: probable carboxylesterase 12. Cyan knots. A0A0D2ZTX3: 14-3-3 protein isoform; A0A0D3A7H6: acyl-coenzyme A oxidase; A0A0D3AFY9: 4-hydroxy-4-methyl-2-oxoglutarate aldolase; A0A0D3AIM5, A0A0D3E2U8, A0A0D3DCR5, and A0A0D3B0N4: malic enzyme isoforms; A0A0D3B0I6: member of the peptidase M3 family; A0A0D2ZUC0: member of the peptidase M16 family; A0A0D3CKD7 and A0A0D3ADS4: member of the peptidase S8 family; A0A0D3C6P9: member of the peptidase S9 family; A0A0D3BEP9: member of the peptidase M3 family; A0A0D3BF46 and A0A0D3DKC4: FBPase domain-containing proteins; A0A0D3C110: acetyl-coenzyme A synthetase; A0A0D3A1W2, A0A0D3B002, A0A0D3BPG1, A0A0D3C6S3, A0A0D3DDK7 and A0A0D3DF71: uncharacterized proteins; A0A0D3C825: acyl-coenzyme A oxidase; A0A0D3ACD4 and A0A0D3BC46: M16C_associated domain-containing proteins; A0A0D3CNS1: lipase 3; A0A0D3CPK6: M16C_associated domain-containing protein; A0A0D3DIE9, A0A0D3DW83 and A0A0D3BJB3: fructose-bisphosphate aldolase isoforms; A0A0D3DNB7: cupin type-1; A0A0D3DR38, A0A0D3DR99 and A0A0D3BD28: aldehyde dehydrogenase isoforms; A0A0D3B358: member of the chaperonin (HSP60) family; A0A0D3B8Y8: dipeptidyl dipeptidase IV; A0A0D3CJL9: jacalin-type lectin; A0A0D3DUP5: transketolase 1; A0A0D3EEX4: oxidored_FMN domain-containing protein; A0A0D3EH74: exonuclease domain-containing protein. Blue knots. A0A0D2ZR55: semialdhyde dehydogenase; A0A0D3A1G6: member of the peptidase M24B family; A0A0D3A267: aminotran_1_2 domain-containing protein; A0A0D3A2C6: glycine cleavage system P protein; A0A0D3A5S1: nucleoside diphosphate kinase; A0A0D3AI31: glutamate dehydrogenase; A0A0D3B8E0: arginase; A0A0D3BAM0: alpha-L-AF_C domain-containing protein; A0A0D3BDK9: aminotran_5 domain-containing protein; A0A0D3BFR4: aldehyde dehydrogenase; A0A0D3BJY4 and A0A0D3CML4: members of the peptidase M24B family; A0A0D3BRB5, A0A0D3C4P3 and A0A0D3DSQ4: phenylalanine ammonia-lyase isoforms; A0A0D3BLL1, A0A0D3BTF3, A0A0D3BTV9, A0A0D3C6Q7, A0A0D3CTX7, A0A0D3D5C6 and A0A0D3AL91: uncharacterized proteins; A0A0D3BXN3: FBPase domain-containing protein; A0A0D3CCT1: spermidine/spermine synthase; A0A0D3CP20: dihydrolipoyl dehydrogenase; A0A0D3E0X8: ligase_CoA domain-containing protein; A0A0D3E680: malate dehydrogenase; A0A0D3EEJ0 and A0A0D3EH53: glutamate dehydrogenase isoforms; A0A0D3EHZ6: glutamine amidotransferase type-2 domain-containing protein; PURA: adenylosuccinate synthetase, chloroplastic.

A detailed description of cargo proteins is a prerequisite to shed light on the functional processes PDNVs are involved in, as well as on their possible effects on mammalian cells. To characterize the protein composition of brassica purified nanovesicles from three biological replicates, a dedicated proteomic analysis was accomplished on pooled 8–11 fractions from anion exchange-FPLC of each preparation. Combined nano-LC-ESI-Q-Orbitrap-MS/MS and bioinformatic analysis identified 345 *B. oleracea* PDNV proteins (Supplementary Table S1), of which about 91% were consistently detected in at least two out of the three biological replicates, and around 66% were shared across all of them. Identified proteins were then analyzed with Mercator software to recognize predominant functional categories. The results showed that identified proteins participate into the following general biological processes: protein homeostasis (25%), photosynthesis (11%), carbohydrate metabolism (8%), amino acid metabolism (6%), lipid metabolism, redox homeostasis and multi-process regulation (5%), nucleotide metabolism (4%), nutrient uptake and phytohormone action (3%), cell wall organization, cell respiration, protein synthesis/modification, and clade-specific metabolism (2%), and secondary metabolism, chromatin organisation, coenzyme metabolism, RNA biosynthesis/processing, DNA damage response, cytoskeleton organisation and vesicle trafficking (1%) (Figure 5a). Other proteins remained not functionally categorized (9%) (Figure 5a). With the aim of studying *B. oleracea* PDNVs proteins according to their association network and functional annotation, the corresponding sequence entries were also analyzed with the STRING suite (Szklarczyk et al., 2023). This analysis allowed predicting a PDNV protein association map including 212 components and consisting of a predominant ramified network linking together 151 molecules, plus 1 distinct quinary and 5 binary molecular complexes (Figure 5b). The involvement of most proteins in the former network emphasized the occurrence of a functional assembly bridging various metabolic pathways and molecular processes. When a functional clustering was applied to this association map by integrating GO categories with Reactome, KEGG, and BioCyc annotations of proteins, 5 groups were identified that included enzymes associated with metabolic processes, and components related to apoplast/secretory vesicles, proteasomal protein processes, protein folding, and intra-intertercellular communication (Figure 5b). Several proteins reported in Supplementary Table S1 were already detected in previous proteomic investigations on PDNVs from various sources. They belonged to different functional classes related to PDNV formation or function, such as cell wall remodelling (α and β galactosidases, UniProtKB codes A0A3P6A8W3 and A03P6CJ23; Jimenez–Jimenez et al., 2019; Woith et al., 2021), proteasome (proteasome subunits α and β, UniProtKB codes A0A0D3C289 and A0A0D3A2X0; Zhang et al., 2016; E1 ubiquitin activating enzyme, UniProtKB code A0A0D3C300; ubiquitin-like protein, UniProtKB code A0A3P6G5J9; Zhang et al., 2016), vescicular trafficking (clathrin, UniProtKB code A0A0D3CL97; annexin, UniProtKB code A0A03P6FKG4; Pocsfalvi et al., 2018a), signaling (14-3-3 protein, UniProtKB code A0A0D3CW05; phospholipase D, UniProtKB code A0A03P6FGA; Pocsfalvi et al., 2018a), oxidative and abiotic stress (catalase, UniProtKB code A0A03P6EYT3; superoxide dismutase, UniProtKB code A0A0D3DTM5; L-ascorbate peroxidase, UniProtKB code A0A03P6DWN0; HSP90, UniProtKB code A0A3P6F4T4; Pocsfalvi et al., 2018a; ferritin, UniProtKB code A0A0D3AZA1; Zhang et al., 2016) and biotic stress (myrosinase, UniProtKB code A0A678XDV3; Rutter and Innes, 2017). Overall, our results indicated that several cargo proteins are specifically enriched in *B. oleracea* PDNVs because they are possibly involved in processes associated with vesicle biogenesis/trafficking and cell-to-cell communication.

A very recent preprint appearing after the submission of the present study has reported the protein repertoire of *B. oleracea* PDNVs obtained through a combination of size exclusion chromatography, trapped ion mobility spectrometry (TIMS)-time of flight (TOF) mass spectrometry and data-independent acquisition (DIA)-parallel accumulation-serial fragmentation acquisition procedures (Rodríguez de Lope et al., 2024). A comparison of the data reported in the present study and those from the above-cited investigation showed a limited overlap of the identified proteins, with a large portion of the assigned components unique to each approach (Supplementary Figure S1). This data discrepancy might likely reflect the significant differences in sample plant source (tissue homogenate vs. apoplastic fluid) as well as in vesicle isolation, protein preparation, mass spectrometry, and proteomic identification procedures used in these studies, with the one described here resolving intact proteins before their trypsinolysis and MS analysis and utilizing stringent identification criteria for molecular assignments. By contrast, the study of Rodríguez de Lope and coworkers avoided fractionating intact proteins before their digestion and MS analysis, used a TIMS-TOF-based DIA acquisition procedure, and did not provide information on the stringency of protein identification parameters, limiting the comparability of results. These differences highlight the importance of the methodological consistency in plant PDNV proteomics and suggest that the experimental approach utilized can substantially influence the definitive characterization of vesicle molecular cargos.

### 3.3 Lipidomic and metabolomic analysis

**FIGURE 6:**
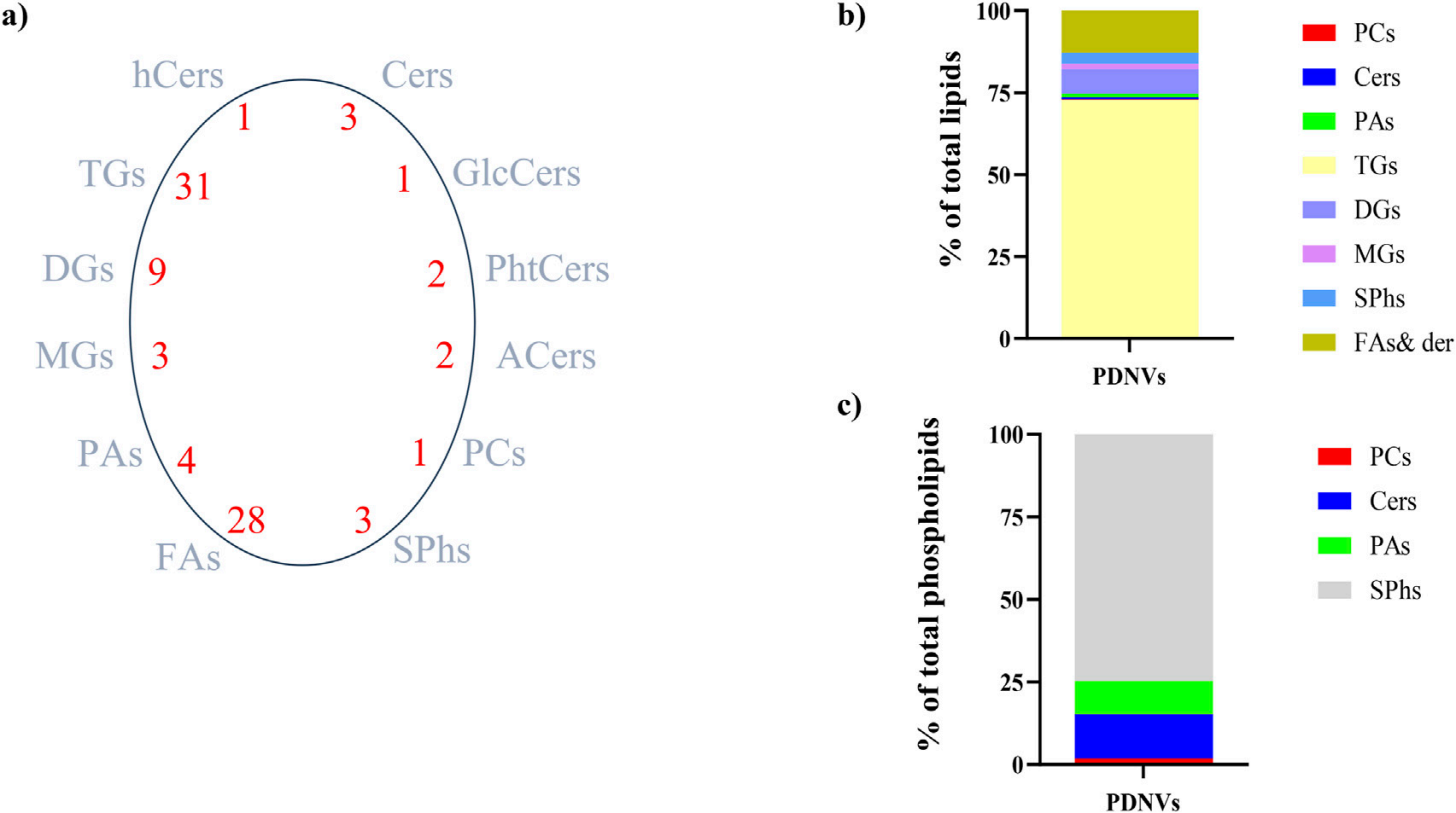
Lipid profile of FPLC-purified *B. oleracea* PDNVs. **(a)** Total lipid species and number of species identified in each lipid group. **(b)** Overall lipid composition, based on area counts. **(c)** Distribution of major types of plasma membrane phospholipids, expressed as a percentage of total phospholipids. Abbreviations: Cers, ceramides; hCers, hydroxyceramides; GlcCers, glucosylceramides; PhtCers, phytoceramides; ACers, acylceramides; PCs, phosphocholines; SPhs, sphinganine and sphingosine; FAs, fatty acids and their derivatives; PAs, phosphatidic acids; MGs, monoalkylglycerols; TGs, triacylglicerols; DGs, diacylglycerols.

Although lipids are essential components of PDNV membranes that influence vesicle stability, biogenesis, and functionality, the study of their composition remains poorly explored. To characterize the lipid profile of anion exchange-FPLC purified PDNVs, total lipids of vesicles were extracted in acid chloroform/methanol and analysed by LC-HR-MS/MS. The results are reported in Figure 6. A total of 88 lipid species, belonging to 12 different classes, were identified (panel a). The most abundant were triacylglycerols, which accounted for almost 75% of total lipids. Diacylglycerols and monoalkylglycerols were also identified as abundant lipids (panel b). From the analysis of the limited available literature, the acylglycerols content of PDNVs from other sources is reduced as compared to brassica PDNVs (Karamanidou and Tsouknidas, 2021). Reasons for this are unclear and could be related to different extraction methods and analysis, or may be linked to the particular acylglycerols-rich profile of brassicaceae plants (Shimada and Hara-Nishimura, 2015). Among structural membrane lipids, sphingolipids (including sphingosine and sphinganine) were the most abundant (60%), followed by phosphatidic acids (20%), phosphatidylcholines (15%) and ceramides (5%), including hydroxiceramides and phytoceramides (panel c). From comparison with literature data, it appears that *B. oleracea* PDNVs had a peculiar lipid profile, significantly different from that of most of other investigated species, which predominantly contain glycerophospholypids, such as phosphatidylcholine, phosphatidic acid, and phosphatidylethanolamine (Ju et al., 2013; Wang et al., 2014; Teng et al., 2018), and to a minor extent, typical plant lipids such as mono- and di-galactosyl diacylglycerols (Zhang et al., 2016; Teng et al., 2018; Zhuang et al., 2015). However, the membrane lipid composition of *B. oleracea* L. PDNVs is fairly in agreement with that determined in *Arabidopsis thaliana,* another member of the *brassicaceae* family (Liu et al., 2020). The complete list of lipids identified in purified *B. oleracea* PDNVs is reported in Supplementary Table S2.

The metabolites present in the acid chloroform/methanol extract were fractionated and identified by an untargeted metabolomics workflow. The results revealed the presence of flavonoids, namely kaempferol and its 3-O-glucoside (astragalin), the phenolic acid sinapinic acid and other polar and non-polar metabolites as amino acid derivatives, organic acids, fatty acid conjugates, vitamins and sugar alcohols (Supplementary Table S3). Along with untargeted workflow, a dedicated procedure for quantification of targeted phytometabolites encompassed the use of analytical reference standards based on matching with tandem MS spectra and with publicly available databases. The calculated concentrations were 37.63 ng mL^−1^ for kaempferol, 21.43 ng mL^−1^ for astragalin, and 5.84 ng mL^−1^ for sinapinic acid. These metabolites are known for their antioxidant and anti-inflammatory properties, which can contribute to the bioactivity of *B. oleracea* PDNVs.

### 3.4 Effect of purified PDNVs on HaCaT cell viability

**FIGURE 7:**
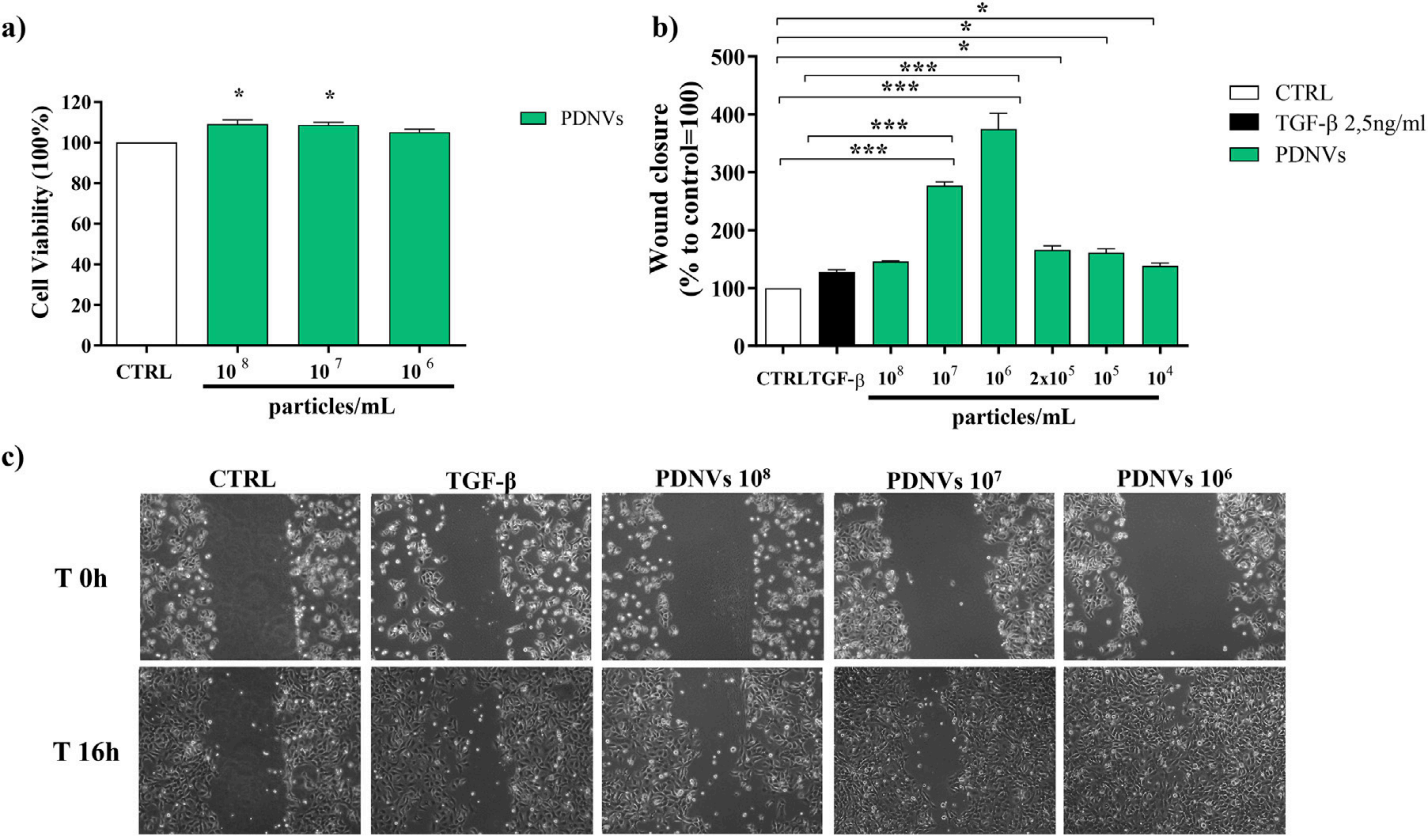
Effect of FPLC-purified *B. oleracea* PDNVs on viability of HaCaT cells **(a)**, and on wound healing in HaCaT cells **(b, c)**. **(a)** PDNVs were administered to HaCaT cells at concentrations of 10^8^, 10^7^, and 10^6^ particles mL^-1^ for 48 h and the corresponding viability was measured. Data are expressed as percentages relative to untreated controls. **(b)** Bar chart showing the percentage of the wound closure effect at the different concentrations used. The percentage of wound closure was estimated using ImageJ software. The non-treated group was used as control. The bars represent the standard deviation values, and asterisks indicate significant differences as determined by one-way ANOVA followed by Tukey’s test (*p < 0.05, **p < 0.01, ***p < 0.001). **(c)** Optical microscopy images of HaCaT cells treated with different PDNVs concentrations, showing the scratched area. Images were captured at 0 and 16 h after PDNVs treatment, using an optical microscopy at ×10 magnification.

In order to evaluate the cytotoxicity of *B. oleracea* PDNVs on human cells, keratinocytes (HaCaT) were treated with different concentrations (10^8^, 10^7^, and 10^6^ particles mL^-1^) of purified PDNVs for 48 h in DMEM. Cytotoxicity was estimated by MTT assay. The results demonstrated that PDNVs were non-cytotoxic at all tested concentrations (Figure 7a).

### 3.5 Effect of purified PDNVs on wound healing in HaCaT cells

The migration of skin cells to the wound site is a pivotal step in wound healing. In order to evaluate the effect of PDNVs on cell migration, HaCaT cells were scratched and incubated with different concentrations of nanovesicles (10^8^, 10^7^, 10^6^, 2 × 10^5^, 10^5^, and 10^4^ particles mL^−1^). Wound closure was measured after 16 h PDNV administration. The results showed that PDNVs promoted wound closure at all tested concentrations (Figure 7b), with the most pronounced effect at 10^6^ particles mL^−1^, in which wound repair was markedly higher than that induced by TGF-β, here used as positive control (Figure 7c). At lower concentrations (10^5^ and 10^4^ particles mL^−1^), the effect although reduced, remained significant compared to the control. These results indicate that PDNVs stimulated human keratinocytes migration, which is a key process in wound repair.

### 3.6 Effect of purified PDNVs on the expression of wound healing-related genes in scratched HaCaT cells

**FIGURE 8:**
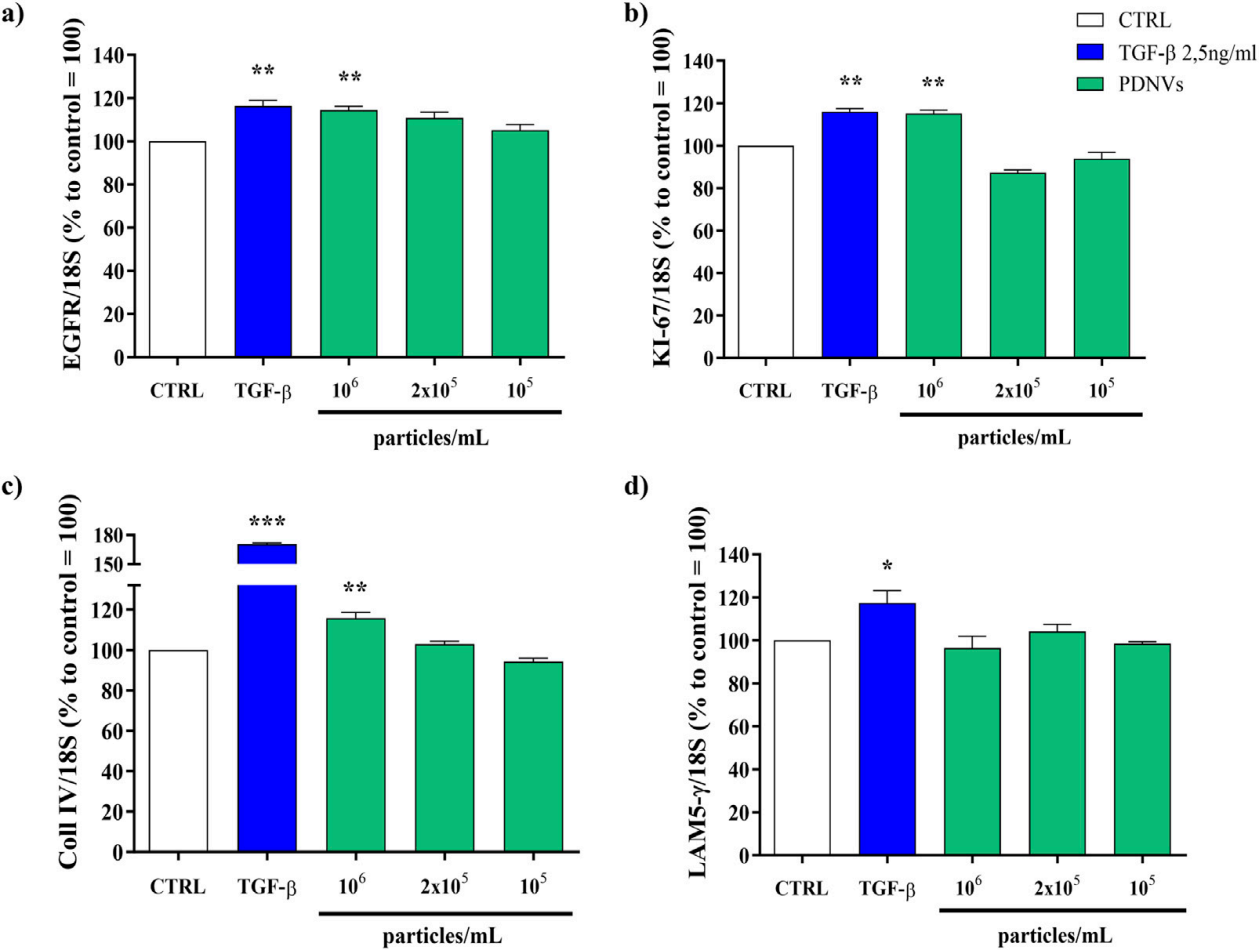
Semi-quantitative RT-PCR gene expression analysis of wound healing-related markers genes in HaCaT cells treated with FPLC-purified *B. oleracea* PDNVs for 16 h, after scratch wound. **(a)** *EGFR* **(b)** *KI-67* **(c)** *Coll IV*, and **(d)** *LAM5-γ* gene expression. Gene expression was determined using semi-quantitative RT-PCR and normalized to the housekeeping gene 18S, with values expressed as a percentage relative to untreated HaCaT cells. Data are presented as relative expression levels, with bars representing standard deviation values, and asterisks indicating significant differences as determined by one-way ANOVA followed by Tukey’s test (*p < 0.05, **p < 0.01, ***p < 0.001).

To investigate the underlying molecular basis of the PDNV wound healing activity, we evaluated by semi-quantitative RT-PCR the expression of different wound healing-associated marker genes in scratched HaCaT cells treated with purified PDNVs. As shown in Figure 8a, PDNV treatment at 10^6^ particles mL^-1^ led to a significant increase of *EGFR* gene expression. The stimulatory effect was comparable to that induced by TGF-β, here used as positive control. In Figure 8b, the effect on the expression of the cell proliferation marker *KI-67* is shown; treatment with a brassica PDNV concentration of 10^6^ particles mL^-1^ induced a 118.00% increase in gene expression, relative to the untreated control, similar to that induced by TGF-β (118.53%). The effects on the extracellular matrix-related genes collagen type IV (*Coll IV*) and laminin subunit 5- γ (*LAM5-γ*), which are involved in cell adhesion and migration, were also evaluated. *Coll IV* expression was significantly upregulated by PDNVs at 10^6^ particles mL^-1^ (Figure 8c), while no significant effect was observed on the expression of the *LAM-5-γ* gene (Figure 8d).

### 3.7 Effect of purified PDNVs on nitric oxide production in LPS-stimulated RAW 264.7 cells

**FIGURE 9:**
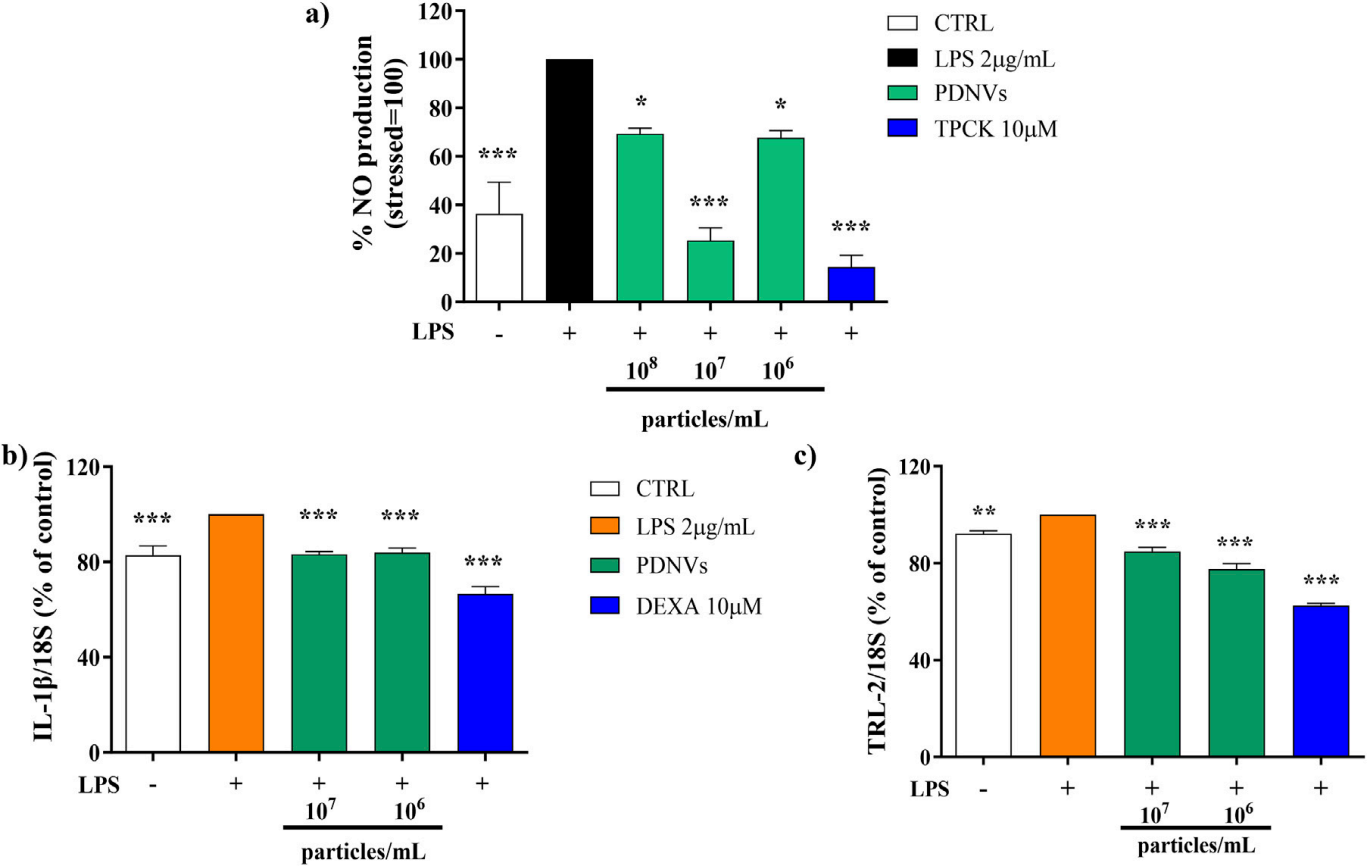
Effect of FPLC-purified *B. oleracea* PDNVs on the inhibition of NO production in LPS-stimulated RAW 264.7 cells **(a)** and on the expression of inflammation-related genes in LPS-stimulated HaCaT cells **(b, c)**. **(a)** Bar chart of NO levels in LPS-stimulated RAW 264.7 cells, pre-treated with PDNVs for 2 h. TPCK, N-tosyl-L-phenylalanine methyl ketone. **(b,c)** Semi-quantitative RT-PCR gene expression analysis of the pro-inflammatory genes *IL-1β* **(b)** and *TLR2* **(c)** in LPS-stimulated, PDNV pre-treated HaCaT cells. The bars represent standard deviation values, and asterisks indicate significant differences as determined by one-way ANOVA followed by Tukey’s test (*p < 0.05, **p < 0.01, ***p < 0.001). Dexa, dexamethasone.

Nitric oxide (NO) production is strongly induced in response to proinflammatory stimuli such as bacterial LPS, a fact that leads to inflammatory citokines and ROS overproduction. In order to evaluate the effect of FPLC-purified brassica PDNVs on cellular NO production under inflammatory conditions, RAW 264.7 cells were cultured with 10^8^, 10^7^, and 10^6^ particles mL^−1^ PDNV concentrations and then stimulated with 2 μg mL^−1^ LPS. After LPS treatment, nitrite levels in the cell culture medium were determined by adding Griess reagent and reading absorbance at 540 nm. As shown in Figure 9a, PDNV pre-treatment significantly reduced the levels of NO, compared to those of PDNV-untreated LPS-stimulated cells. Specifically, 10^7^ particles mL^-1^ concentration induced a 75% reduction of NO production, an effect comparable to that of TPCK, a serine protease inhibitor known to reduce inflammation by blocking NF-κB activation.

### 3.8 Effect of purified PDNVs on the expression of inflammation-related genes in LPS-stimulated HaCaT cells

The effect of FPLC-purified *B. oleracea* PDNVs on the expression of inflammatory genes in HaCaT cells pre-incubated with different concentrations of PDNVs (10^7^ and 10^6^ particles mL^−1^), and treated with LPS (2 μg/mL) was evaluated by semi-quantitative RT-PCR. The aim was to assess the potential of PDNVs to downregulate the expression of pro-inflammatory cytokines, such as interleukin-1β (*IL-1β*), and the toll-like receptor 2 (*TLR-2*). The results showed that the pre-treatment with PDNVs downregulated the expression of *IL-1β* compared to LPS-induced cells (Figure 9b), to the same extent at both tested concentrations. The effect was lower as compared to that induced by the anti-inflammatory positive control DEXA (at 10 μM). On the other hand, a more pronounced and dose-dependent downregulation of the expression of the *TLR-2* was observed (Figure 9c).

### 3.9 MicroRNA analysis and computational prediction of putative human target genes

**FIGURE 10:**
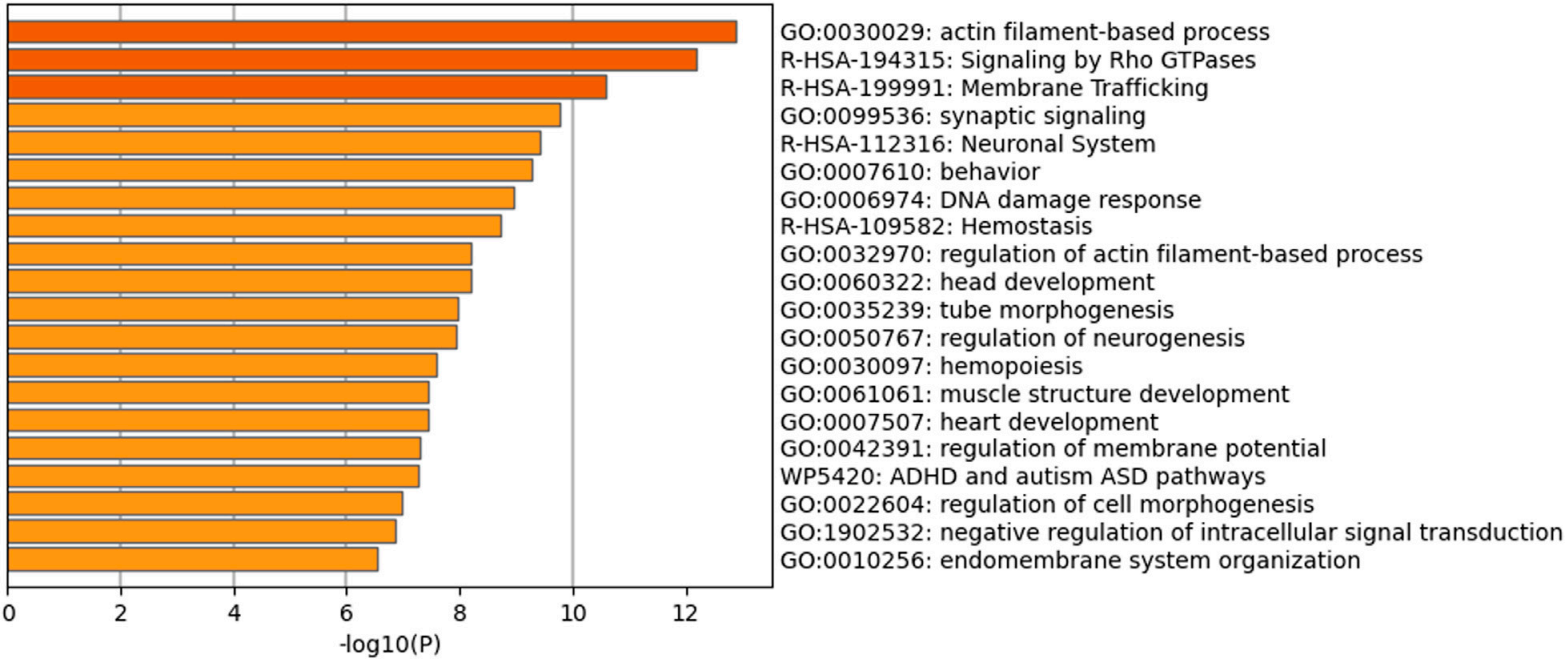
Functional enrichment analysis based on all the predicted human target genes for the whole set of brassica miRNAs identified in PDNVs. The bar chart contains Gene Ontology (GO) terms and Wiki and Reactome pathways resulted by the enrichment analysis. Biological processes and pathways were ordered according to their p-value, from the most significant (dark orange) to the lowest one (light orange), [x-axis: -log10 (p-value); y-axis: enriched terms].

PDNVs have emerged as mediators of interspecies communication, and growing evidence suggests their participation in the regulation of mammalian gene expression (Mu et al., 2023). To explore this potential, we analysed the miRNA profile of *B. oleracea* PDNVs. The miRNAs were purified from total RNA isolated from FPLC-purified PDNVs and subjected to high-throughput sequencing. The miRNA expression profile was obtained by mapping to the reference database PmiREN2.0. A total of 150 miRNAs were identified, 24 of which corresponded to unique sequences, which are reported in Table 1 together with the respective count readings. The complete list of identified miRNAs is reported in Supplementary Table S4. In order to shed light on the potential involvement in the regulation of specific cellular processes, a bioinformatic analysis using the psRNAtargets algorithm was carried out to predict potential human genes targeted by the identified miRNAs. The most likely candidate for each miRNA, based on expectation values, is reported in Table 2, whereas the complete list of predicted target genes is reported in Supplementary Table S5. The results showed that 16 putative target genes were identified as potentially regulated by brassica miRNAs. Overall, they indicate the potential of *B. oleracea* miRNAs to target a number of human genes associated with key biological processes relevant to tissue repair and inflammation, including extracellular matrix organization, cell cycle regulation, and cytokine signaling. To further elucidate the biological significance of the identified target genes, an enrichment analysis was conducted using Gene Ontology (GO) and KEGG annotations of cellular process. GO/KEGG terms were selected based on accumulative hypergeometric p-values and enrichment factors. Hierarchical clustering was performed using a kappa score threshold of 0.3, with the results visualized as a network in Cytoscape. In Figure 10, the bar chart of GO/KEGG pathway enrichment analysis for predicted human target genes is reported. Among these pathways, actin filament-based process showed the highest score of enrichment. Actin-based motility is crucial in cell migration, adhesion, and structural organization. The second and third most enriched pathways were signalling by Rho GTPases and membrane trafficking, which are essential for intracellular transport, communication, and cytoskeletal dynamics, which are processes required for cellular migration and tissue remodelling. Furthermore, the enrichment of pathways related to response to injury and stress (hemostasis and DNA damage response) or to structure development and morphogenesis (heart development, head development, muscle structure development, tube morphogenesis, cell morphogenesis), suggests the potential function of brassica-derived miRNAs in regulating pathways associated with cell differentiation and tissue organization, key processes for tissue regeneration and repair.

**TABLE 1:** miRNA profile of FPLC-purified *B. oleracea* PDNVs. The PmiREN2.0 reference database, including sequences from *B. oleracea* (Bol), *B. juncea* (Bju), *B. napus* (Bna), *B. nigra* (Bni) and *B. rapa* (Bra) was used for analysis. Asterisks indicate sequences that are complementary, opposite strand sequences to miRNA.

miRNA name	miRNA 5’-3’sequence	High quality reads
Bna-miRN289*	GTG​GCG​AGA​GCG​GAC​CGC​CTC	573551
Bna-miRN289	GGC​GGG​CTG​CTT​GAG​CTG​CA	80149
Bra-miRN351	GCT​TGT​GGT​TCG​GAC​ACT​CT	16030
Bra-miRN357a	TGC​GGA​AGG​ATC​ATT​GTC​GTA	9727
Bju-miR319b	TTG​GAC​TGA​AGG​GAG​CTC​CTT	3433
Bra-miR319d	TTG​GAC​TGA​AGG​GAG​CTC​CCT	3433
Bna-miR319b	TTG​GAC​TGA​AGG​GAG​CTC​CC	3433
Bna-miR156e	TGA​CAG​AAG​AGA​GTG​AGC​ACT	2181
Bju-miR156x	TGA​CAG​AAG​AGA​GTG​AGC​ACA	2151
Bju-miR156x	GAC​AGA​AGA​GAG​TGA​GCA​CA	2151
Bni-miR156a	TGA​CAG​AAG​AGA​GTG​AGC​AC	2139
Bju-miR156j	GTG​ACA​GAA​GAG​AGT​GAG​CAC	2139
Bna-miR156i	TTG​ACA​GAA​GAG​AGT​GAG​CAC	2126
Bju-miR156ad	CTG​ACA​GAA​GAG​AGT​GAG​CAC	2111
Bna-miR156p	GAC​AGA​AGA​GAG​TGA​GCA​CAT	2102
Bna-miR157j	TTG​ACA​GAA​GAT​AGA​GAG​CAC	2094
Bol-miR157	TTG​ACA​GAA​GAT​AGA​GAG​CAC​T	2094
Bju-miR168f	TCG​CTT​GGT​GCA​GGT​CGG​GAC	1954
Bol-miR168b	TCG​CTT​GGT​GCA​GGT​CGG​GAC​T	1951
Bna-miR168r	TCG​CTT​GGT​GCA​GGT​CGG​GAA	1951
Bna-miR168t	TCG​CTT​GGT​GCA​GGT​CGG​GA	1951
Bra-miR168f	TCG​CTT​GGT​GCA​GGT​CGG​GAA​C	1914
Bju-miR156y	CGA​CAG​AAG​AGA​GTG​AGC​AC	1236
Bra-miRN357b*	TAA​CAA​GGT​TTC​CGT​AGG​TGA	1114

**TABLE 2:** List of putative human target genes predicted for the miRNAs identified in brassica PDNVs.

miRNA	Target gene	Expectation value	Biological function	Gene aligned sequence
Bna-miRN289	NM_152830|ACE	2.5	Protein Angiotensin-Converting Enzyme; key role in the renin-angiotensin system; regulates blood pressure and electrolyte balance	UCC​AGC​CCA​GGC​AGC​CCG​CC
Bna-miR319b	NM_014906|PPM1E	2	Protein Phosphatase, Mg^2+^/Mn^2+^ Dependent 1E, negative regulator of kinase activity, influencing cellular processes such as cytoskeletal remodelling and metabolic regulation	AGU​AGU​UCC​CUU​CAG​UUC​AA
Bju-miR156x	NM_152795|HIF3A	2.5	Hypoxia-Inducible Factor 3 Alpha Subunit; involved in the adaptive response to hypoxia and suppresses hypoxia-inducible gene expression	ACU​GCU​CGC​UCU​GUU​UUG​UCA
Bju-miR156x	NM_152387|KCTD18	2.5	Potassium Channel Tetramerization Domain Containing 18; essential roles in proliferation, differentiation, apoptosis, and metabolism	UUU​UCU​UAC​UCU​UUU​CUG​UUA
Bni-miR156a	NM_001178097|C12orf74	2.5	Pleckstrin Homology And RhoGEF Domain Containing G7; involved in Rho protein signal transduction	UCG​CUC​ACU​UUC​UUC​UGU​CC
Bna-miR156i	NM_033121|ANKRD13A	2	Ankyrin Repeat Domain 13A; involved in various cellular processes, including signal transduction, cell cycle regulation, and cytoskeletal organization	UAG​UUA​ACU​CUC​UUU​UGU​CAA
Bna-miR156i	NM_001143943|EFCAB2	2.5	EF-hand Calcium Binding Domain 2; involved in calcium ion binding	UAG​UUC​AUU​CUU​UUU​UGU​UAA
Bna-miR156i	NM_004674|ASH2L	2.5	ASH2-like, histone lysine methyltransferase complex subunit; plays a crucial role in the regulation of gene expression	CAG​GUC​CCU​CUU​UUC​UGU​CAA
Bju-miR156i	NM_152510|HORMAD2	2.5	HORMA Domain Containing 2; involved in chromatin dynamics and DNA repair processes, meiotic progression and the maintenance of genome stability	UUG​CUC​AAU​UUU​UUU​UGU​CAG
Bju-miR156ad	NM_001080414|CCDC88C	2.5	Coiled-Coil Domain Containing 88C, also known as Daple; involved in intracellular signalling cascades, particularly in the Wnt signalling pathway, and cell migration and adhesion	GGA​GUC​ACU​CUC​UUC​UGU​CGG
Bna-miR156p	NM_016946|F11R	2	Junctional Adhesion Molecule A (JAM-A), member of the immunoglobulin superfamily and plays a role in maintaining cellular junction integrity, regulating immune cell movement, and modulating platelet function	UGG​GGC​CCA​CUC​UCU​UCU​GUC
Bju-miR157a	NM_001031848|SERPINB8	1.5	Serpin B8: member of the serine protease inhibitor (serpin) family; regulates inflammation and immune responses	AUG​CUC​UUU​GUC​UUU​UGU​CAA
Bju-miR157a	NM_007021|C10orf10	2	DEPP1 (Decidual Protein Induced by Progesterone); acts as a critical modulator of FOXO3-induced autophagy via increased cellular reactive oxygen species (ROS)	AUG​UUU​UCU​GUC​UUC​UGU​UAA
Bju-miR168f	NM_145166|ZBTB47	2	Protein predicted to function as a DNA-binding transcription factor, specifically RNA polymerase II-specific, involved in the regulation of transcription	UCC​CUG​ACC​UGC​ACC​AGG​UGG
Bju-miR156y	NM_001256272|VSX1	2.5	Visual System Homeobox 1, involved in the regulation of cone opsin genes during early development	UUU​UUC​ACU​UUC​UUC​UGU​UG
Bju-miR156y	NM_005737|ARL4C	2.5	ADP-ribosylation factor-like protein 4C, involved in cellular processes such as vesicular trafficking and cell proliferation	CUG​CUU​UUU​CUC​UUU​UGU​CG

For each miRNA, the best target was reported in form of NCBI, reference sequence/gene official symbol, together with the relative expectation value, description of biological function and gene aligned sequence. The results showed that 16 putative target genes were identified as potentially regulated by brassica miRNAs. overall, they indicate the potential of *B. oleracea* miRNAs, to target various human genes associated with key biological processes relevant to tissue repair and inflammation, including extracellular matrix organization, cell cycle regulation, and cytokine signaling.

## 4 Discussion

Although in recent years PDNVs have been the subject of ever-growing interest because of their multiple therapeutic potential applications, the main challenge to their exploitation (and study) remains the availability of a cost-effective procedure ensuring large scale production of highly purified vesicles. To overcome these challenges, we developed a purification platform that integrates ultrafiltration with FPLC anion exchange chromatography and that can be easily scaled up. The effectiveness of this procedure was tested by applying it to the purification of PDNVs from *B. oleracea* L*.,* a plant worldwide cultivated and appreciated for its nutritional characteristics and already demonstrated as a valid source of vesicles with interesting biological properties (You et al., 2021). The results have demonstrated that our platform produced a large amount of concentrated (2,2 × 10^10^ particles mL^−1^), homogeneous, and pure PDNVs. NTA analysis showed that the FPLC step was decisive, removing 90% of high molecular mass contaminants, such as protein aggregates and nucleosomes, and producing a homogeneous population of small PDNVs (mean size 138.2 ± 1.5 nm) with a purity index comparable to those of vesicles purified from animal fluids, where much less contaminants are present (Kim and Shin, 2021; Gagnon et al., 2020). TEM morphological analysis proved that the FPLC-purified *B. oleracea* PDNVs were structurally intact, a pre-requisite for activity, retaining their physiological spherical shape, with a well-defined boundary between the lipid bilayer membrane and the surrounding medium. Exploitation of PDNVs also suffers from the relatively scarce characterization of their structural components as well as of their cargo molecules, whose improved knowledge could lead to more focussed applications as well as to expand their range. Therefore, a systematic and thorough analysis of the molecular cargos of FPLC-purified brassica PDNVs was performed using omic approaches. Proteomic analysis identified 345 proteins, and functional GO and KEGG enrichment analysis showed that some of them were grouped into functional classes related to the formation and transport of PDNVs, including proteasomal protein processing and apoplast/secretory vesicle trafficking, or related to PDNV functions, including cell wall remodelling, intercellular communication, and molecular signalling. Our study confirmed the enrichment in specific functional classes observed in previous studies on PDNVs from different sources (Zhang et al., 2016; Ju et al., 2013; Jimenez-Jimenez et al., 2019; Pocsfalvi et al., 2018a; Pocsfalvi et al., 2018b; Urzì et al., 2021; Rutter and Innes, 2017; Rodriguez de Lope, 2024). Currently, information about the involvement of cargo proteins in the modulation of specific processes or pathways in host cells is very poor, limited to a few but significant cases. For example, it has been shown that blocking the mannose-binding lectin II of garlic PDNVs hampers their endocytosis in HepG2 cells (Song et al., 2020) or that heat shock protein (HSPA8) from mulberry bark PDNVs binds to and activates the aryl hydrocarbon receptor pathway, with a reduction in the inflammatory response in drug-induced colitis (Sriwastva et al., 2022). These pieces of evidence demonstrate that the precise identification of conserved PDNV cargo proteins is a prerequisite for understanding their biochemical functions in host cells that underly PDNV activities in plant and mammalian cells, and for paving the way to their manipulation for therapeutic applications.

Although PDNVs are membrane-enclosed nanovesicles whose composition can affect stability, transport, cell recognition, and signalling, systematic information on their lipid composition is very poor. Lipidomic analysis of *B. oleracea* PDNVs revealed a peculiar composition of membrane lipids, which are of major importance for biological functions. Sphingolipids, including sphingosine and sphinganine, as well as various types of ceramides, were the most abundant, followed by phosphatidic acid. This composition differed significantly from those of PDNVs from other sources up to now investigated, where phospholipids (phosphatidic acid, phosphatidylcholine, and phosphatidylethanolamine) and mono- and digalactosylglycerols were the most abundant species (Karamanidou and Tsouknidas, 2021). Howerver, these data are consistent with those from *A. thaliana,* another member of the brassicaceae family, where sphingolipids constituted approximately 46% of total lipids (Liu et al., 2020). The lipid content has been shown to modulate PDNV targeting and uptake, as well as to have repair and anti-inflammatory properties in mammalian tissues and organs (Chen et al., 2023). In this sense, available data refer primarily to phospholipids for targeting and repair (Teng et al., 2018; Xu et al., 2022) and to digalactosydiacylglycerols for anti-inflammatory properties (Xu et al., 2022), while data for sphingolipids are lacking. On the other hand, sphingolipids in mammalian cells are important modulators of cell functions, including proliferation, apoptosis, migration, and inflammatory response, by regulating pro-inflammatory transcription factors and prostaglandin production (Wasserman et al., 2020; Nixon, 2009). Interestingly, You and coworkers demonstrated that brassica-purified PDNVs promoted cell proliferation and suppressed inflammation and apoptosis in mammalian cells (You et al., 2021). These data suggest that sphingolipids, in addition to phospholipids, can act as modulators of target cell functions, and open the way to more oriented studies of the biochemical pathways underlying PDNV effects in target cells and to the modification of the PDNV lipid composition, for therapeutic applications.

Metabolomic analysis revealed the presence in purified brassica PDNVs of the flavonoid kaempferol and its 3-O-glucoside astragalin, and of the phenypropanoid sinapinic acid, together with many other primary metabolites. From the few metabolomic studies performed up to now on PDNVs, it has been suggested that the secondary metabolites contained in PDNVs can contribute to their biological and pharmacological activities. Flavonoids are a class of well-known antioxidant and anti-inflammatory compounds. In grapefruit PDNVs, the flavonoids naringin and naringenin were detected and potentially related to the protective effect of grapefruit PDNVs on mouse colitis (Wang et al., 2014). The phenylpropanoids shogaol and gingerol were identified in ginger PDNVs by Zhuang et al. (2015), who also demonstrated that shogaol in ginger PDNVs had a liver protective effect by activating the NrF2 pathway. Interestingly, the p-hydroxycinnamic acid sinapinic acid and the corresponding esters are highly accumulated in all organs of plants of the brassicaceae family, where they perform an adapataive role (Nguyen et al., 2021). They are attracting increasing interest due to their remarkable antioxidant and anti-inflammatory activities and potential pharmaceutical applications. However, it has been observed that the accumulation of secondary metabolites in PDNVs could be unspecific, and their recovery in purified vesicles variable in dependence on the extraction conditions (for example, pH) (Woith et al., 2021), so that much further work is still necessary to fully demonstrate if secondary metabolites contribute effectively to the biological properties of PDNVs.

To test the bioactivity of FPLC-purified brassica PDNVs, their skin repair and anti-inflammatory properties were assayed *in vitro* on human cells. The results of wound healing assay on HaCaT cells demonstrated that these PDNVs were quite effective in promoting wound closure in scratched cells, underliyng their ability to stimulate cell migration and proliferation. Semi-quantitative RT-PCR analysis showed a positive correlation with upregulation of skin regeneration-related genes such as *EGFR*, *KI-67*, *Coll IV* and *LAM5-γ*, which are critical for cell proliferation, extracellular matrix remodelling, and cellular adhesion, respectively, during the healing process. PDNVs from *B. oleracea*, purified by a different protocol, have been shown to stimulate the proliferation of HaCaT and RAW 264.7 cells at similar concentrations (You et al., 2021); stimulation of cell proliferation was already associated with PDNVs from various sources, such as aloe PDNVs in human fibroblasts (Kim and Park, 2022), bitter melon PDNVs in cardiomyocytes H9C2 cells (Cui et al., 2022) and grape PDNVs in intestinal epithelial stem cells (Ju et al., 2013). The underlied molecular pathways (s) are currently unknown, although in the case of epithelial stem cells the Wnt/β catenin signalling pathway resulted stimulated (Ju et al., 2013). Regarding cell migration, a key process in wound healing, the stimulatory potential of PDNVs has already been reported, particularly with aloe PDNVs in HaCaT and HDF cells (Sánchez-López et al., 2022; Kim et al., 2021). Furthermore, wheat PDNVs have been shown to stimulate fibroblast migration and to induce the secretion of collagen I, a key protein of the cellular matrix necessary for cell migration (Şahin et al., 2019). This latter finding appears to be positively correlated with the observed upregulation of the *Coll IV* gene under our conditions. The NO production assay in RAW.264.7 showed that PDNVs reduced NO levels under inflammatory-LPS stimulation, thus demonstrating their anti-inflammatory potential. This finding was supported by semi-quantitative RT-PCR analysis of pro-inflammatory gene expression in HaCaT cells, which showed that PDNVs downregulated the transcription of the pro-inflammatory cytokine *IL-1β* gene and of the Toll-like receptor-2 gene (*TLR-2*). Our data are in accordance with those reported by You and coworkers, who showed that brassica PDNVs reduced the transcription of pro-inflammatory *IL-1β*, *IL-6* and *COX-2* genes in LPS-stimulated RAW264.7 cells (You et al., 2021).

A growing body of evidence suggests that miRNAs contained in PDNVs are absorbed and regulate gene expression in mammals (Mu et al., 2014; Karamanidou and Tsouknidas, 2021; Subha et al., 2023; Urzì et al., 2021), including genes involved in the anti-inflammatory response (Xiao et al., 2018), and in tissue regeneration (Chen et al., 2023). To shed light on the molecular basis underlying the effects on inflammation, and cell migration/proliferation observed *in vitro*, we first identified the miRNA population present in purified *B. oleracea* PDNVs and subsequently carried out a bioinformatic analysis to identify human genes potentially targeted by these miRNAs. Results showed that among those identified, different miRNAs have already been described for their anti-inflammatory and regenerative properties. MiR156a from blueberry PDNVs, has been shown to exert a protective effect on human endothelial cells by modulating their response to TNF-α (De Robertis et al., 2020). MiR319d from blueberry PDNVs has been related to a protective role in inflammation (De Robertis et al., 2020). MiR156d from bitter melon PDNVs has been shown to potentially regulate NLRP mRNA, which plays a key role in the regulation of inflammatory pathways (Feng et al., 2018). Interestingly, brassica PDNVs contained also miR168, which has been reported to reduce inflammation mediated by TLR agonists, through a TLR3-dependent mechanism. This miRNA from *Fragaria vesca* has been shown to suppress the inflammatory response by decreasing the expression of the TRIF transcript, an essential adaptor protein required for innate TLR3-mediated immune responses (Kameli et al., 2021). These findings suggest that miR168 could play a central role in mitigating inflammatory processes and contribute to the overall anti-inflammatory properties of PDNVs.

A bioinformatic analysis to predict human genes potentially targeted by the identified miRNAs was also carried out. The analysis identified 16 putative best target genes. Seven of them were associated with processes crucial for tissue repair. Specifically, two genes were implicated in cytoskeletal organization and remodelling (*PPM1E* and *ARL4C*), one in cell proliferation (*KCTD18*), one in cell migration and adhesion (*F11R/JAM-A*), and one in cell movement (*ANKRD13A*). One gene, *SERPINB8*, was associated with anti-inflammatory responses, specifically inflammation and immune regulation, while another gene (*C10orf10/DEPP1*) was related to autophagy in response to oxidative stress. Overall, results from miRNA profile analysis confirmed the presence in brassica PDNVs of various miRNAs that may be hypothetically associated with the regulation of human anti-inflammatory and wound healing pathways, and identified new molecules potentially involved in the regulation of crucial processes for tissue repair and inflammation inhibition, thus providing a rationale for their observed *in vitro* effects, useful to address future research and applications.

In conclusion, in this work we have developed an anion exchange-based method for the isolation and purification of extracellular vesicles from plant sources, which is highly efficient in terms of purity and yield as well as easy scalable, for therapeutic and industrial applications. Our approach, providing high pure PDNVs, allowed an extensive and confident characterization of their molecular content through proteomics, lipidomics, metabolomics and miRNomics. Molecular analyses led to the identification of different classes of signalling molecules, such as sphingolipids and miRNAs that can account for the *in vitro* tissue repair and anti-inflammatory properties of *B. oleracea* PDNVs and helpful to orient new studies about their mechanism(s) of action and future applications.

## Data Availability

The mass spectrometry proteomics data have been deposited to the ProteomeXchange Consortium via the PRIDE partner repository with the dataset identifier PXD063392.
