# Supplementary figures and images for: Anion exchange chromatography-based purification of plant-derived nanovesicles from Brassica oleracea L.: molecular profiling and bioactivity in human cells
Source: Front Bioeng Biotechnol. 2025 Jul 31;13:1617478. doi: 10.3389/fbioe.2025.1617478 (PMC12350333; doi:10.3389/fbioe.2025.1617478)

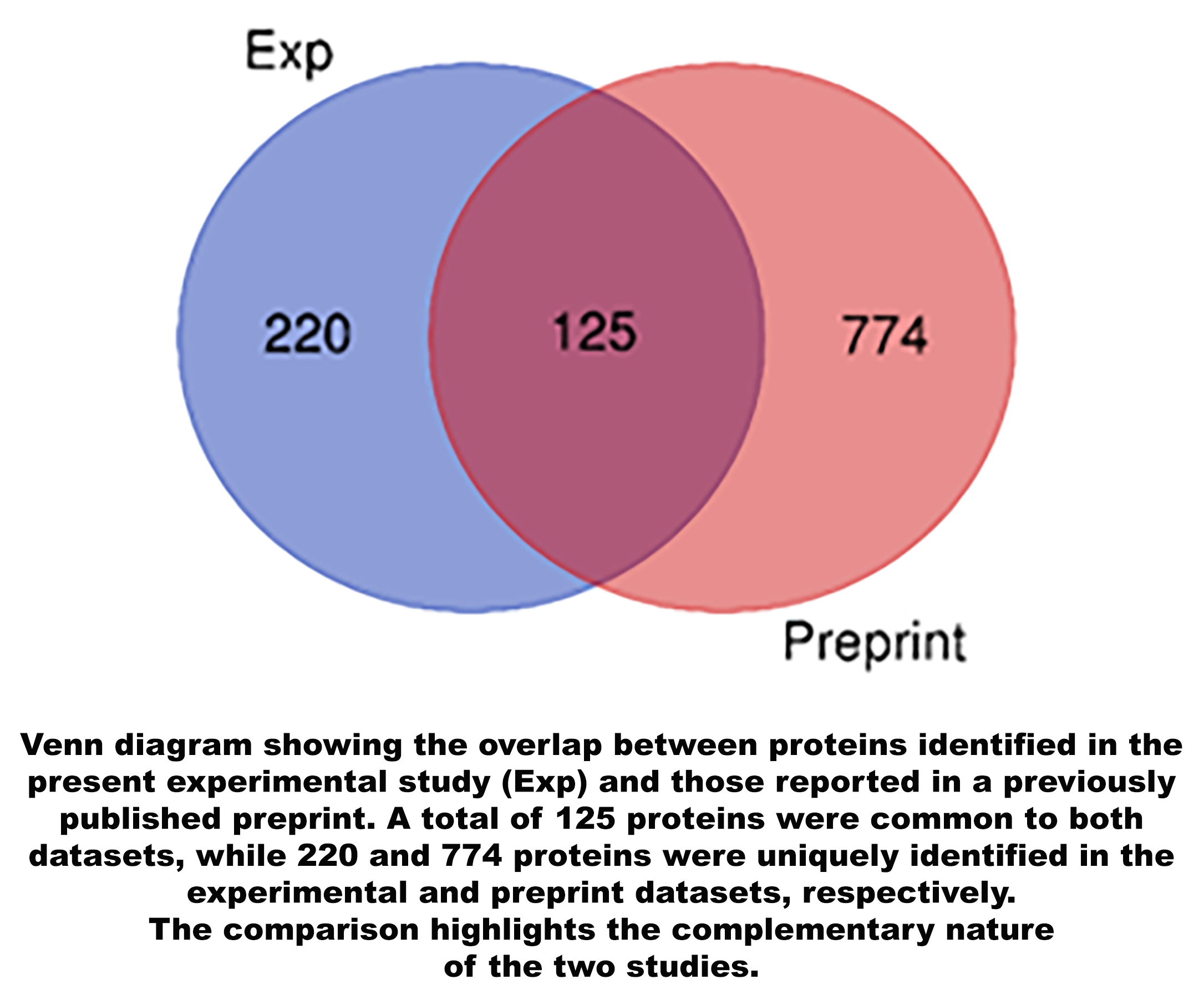

Supplement: Supplementary file 6 [file Image1.jpg]
